# Engagement in Hypertension and Diabetes Clinical Trials at Federally Qualified Health Centers: A Systematic Review

**DOI:** 10.1001/jamanetworkopen.2025.5258

**Published:** 2025-04-15

**Authors:** Samuel Byiringiro, Rifath Ara Alam Barsha, Thomas Hinneh, Emmanuel Uwiringiyimana, Juliana K. Garcia, Kimesha Grant, Tosin Tomiwa, Khadijat Adeleye, Brenda Owusu, Yuling Chen, Diana-Lyn Baptiste, Ashwag Alhabodal, Serina Gbaba, Payam Sheikhattari, Hailey N. Miller, Anna Steeves-Reece, Anna Templeton, Cheryl R. Dennison Himmelfarb

**Affiliations:** 1 Johns Hopkins School of Nursing, Baltimore, Maryland; 2 Johns Hopkins School of Medicine, Baltimore, Maryland; 3 College of Medicine and Health Sciences, University of Rwanda, Kigali; 4 Elson S. Floyd College of Medicine, Washington State University, Spokane; 5 Elaine Marieb College of Nursing, University of Massachusetts, Amherst; 6 University of Miami School of Nursing & Health Studies, Miami, Florida; 7 School of Nursing, University of Connecticut, Storrs; 8 School of Community Health and Policy, Morgan State University, Baltimore, Maryland; 9 OCHIN Inc, Portland, Oregon; 10 John Hopkins Bloomberg School of Public Health, Baltimore, Maryland

## Abstract

**Importance:**

Federally qualified community health centers (FQHCs) are potential partners in the quest to increase diversity in clinical trials. Despite this opportunity, there is limited knowledge about FQHC engagement in clinical trials.

**Objective:**

To assess levels of FQHC engagement in hypertension and type 2 diabetes (T2D) clinical trials and identify FQHC characteristics associated with engagement in the US.

**Evidence Review:**

Six literature databases were searched for protocols and reports of clinical trials addressing hypertension or T2D among adults at FQHCs in the US, published between January 1, 2013, and November 6, 2023. Guided by a framework on community-engaged research, 4 levels of FQHC engagement in clinical trials were defined, ranging from level 1 (FQHC informed) to level 4 (FQHC driven). An ordinal regression analysis was conducted to investigate the association between FQHC organizational and patient demographic characteristics and levels of engagement in hypertension and T2D clinical trials using the publicly available data from Uniform Data System (UDS) for all identifiable FQHCs.

**Findings:**

The initial literature search identified 4552 articles. Following deduplication, title and abstract screening, full-text review, data extraction, and matching with available information in UDS, a total of 33 clinical trials were included. Together, these clinical trials engaged 67 FQHCs. In most cases, FQHC engagement occurred at level 1 (15 clinical trials engaging 19 FQHCs) or level 2 (8 clinical trials engaging 38 FQHCs). A higher ratio of full-time equivalent physicians to patients was associated with 54% (odds ratio [OR], 1.54; 95% CI, 1.06-2.23) higher odds of having a higher level of FQHC engagement in hypertension and T2D clinical trials. A higher ratio of full-time community and patient education specialists to patients was associated with 41% (OR, 1.41; 95% CI, 1.03-1.94) higher odds of having a higher level of FQHC engagement in hypertension and T2D clinical trials.

**Conclusions and Relevance:**

In this systematic review of FQHC engagement in clinical trials, lower levels of engagement in hypertension and T2D clinical trials were found. Further research is required to identify clinical trial design and implementation strategies that promote FQHC participation in clinical trials and research capacity building.

## Key Points

**Question:**

What are the engagement levels of federally qualified community health centers (FQHCs) in hypertension and type 2 diabetes clinical trials, and what FQHC characteristics are associated with levels of engagement?

**Findings:**

In this systematic review including 33 clinical trials, 23 engaged with 57 of 65 FQHCs identified from the uniform data system at the lower levels of engagement. Higher numbers of physicians and community and patient education specialists were associated with higher odds of upper levels of engagement.

**Meaning:**

These findings suggest that FQHCs make minimal contributions to the design and conduct of clinical trials, which limits their ability to build capacity for research conduct and involvement.

## Teaser

This systematic review assesses the current levels of engagement by federally qualified health centers in hypertension and type 2 diabetes clinical trials in the US and identifies health center characteristics associated with engagement.

## Introduction

The lack of diversity in clinical trials hinders equitable access to the benefits of medical advancements, especially in cardiovascular disease (CVD) research. Despite higher rates of cardiometabolic diseases like hypertension and type 2 diabetes (T2D) among underserved populations, including American Indian or Alaska Native, Black, Latino, and uninsured populations, these groups lack meaningful representation in CVD trials.^1,2,3,4^ For example, Black adults made up only 3% of participants in clinical trials for 24 major CVD drugs approved by the US Food and Drug Administration from 2006 to 2020, despite representing 13% of the US population.^4,5^ To fully realize the potential of medical science, greater efforts are needed to include historically underrepresented populations in clinical trials.

Federally qualified community health centers (FQHCs) offer a valuable opportunity to boost participation of underrepresented populations in clinical trials. With approximately 1500 FQHCs and 14 000 service sites across the US, FQHCs serve 31 million US residents, 65% of whom are from racial and ethnic minority groups.^6,7^ These centers provide trusted health care to low-income, uninsured, and rural populations, making them well positioned to overcome barriers to trial participation. Enhancing FQHC engagement in clinical trials could assist in effectively recruiting underserved populations, helping to diversify research efforts.

There is lack of understanding of the status of FQHC engagement in clinical trials and the challenges they face in participating. A decade-old survey of 386 FQHCs found that 56% were involved in some form of research, although not always in clinical trials, and more than half of those not engaged expressed interest in participating.^8,9^ Barriers to engagement include lack of dedicated research staff, concerns over reduced clinical productivity, insufficient research training, and limited eligible funding.^9^ This systematic review and secondary data analysis assessed the current levels of FQHC engagement in hypertension and T2D clinical trials in the US and identified FQHC characteristics associated with engagement.

## Methods

### Protocol and Registration

In conducting this systematic review, we followed the Preferred Reporting Items for Systematic Reviews and Meta-Analyses (PRISMA) reporting guideline. The protocol of the current systematic review was registered on Prospero (CRD42023453760).

### Search Strategy

We searched PubMed, Cochrane, CINAHL (Cumulative Index of Nursing and Allied Health Literature), Web of Science, Embase, and Scopus for studies conducted between January 1, 2013, and November 6, 2023. A public health informationist supported the team in the development of the search strategy. The key concepts of our search strategy included FQHCs (in full name) and look-alikes, randomized clinical trials (RCTs), hypertension, and T2D. Alternate names used to refer to FQHCs included rural, urban, neighborhood, tribal, federally qualified, nurse-managed, and migrant health centers or clinics. A complete search strategy is provided in eMethods 1 in Supplement 1.

### Eligibility Criteria

We included reports or protocols of RCTs that (1) addressed hypertension or T2D among adults 18 years or older, (2) involved all types of medical interventions, (3) engaged 1 or multiple FQHCs or look-alikes (similar institutions that are not currently receiving funding from the government) with or without other non-FQHC sites in the US, and (4) were published in 2013 and beyond and in English (eTable 1 in Supplement 1). We limited the search to 2013 and beyond because of major policy changes that affected the funding and operations of FQHCs in the last 10 years.^10^ We excluded observational and nonrandomized interventional studies and gray literature (ie, nonacademic reports, white papers, and opinions).

### Study Selection

We imported all identified articles to EndNote to remove duplicates and then uploaded them to Covidence, a systematic review management software.^11^ Additional duplicates were identified and removed by Covidence. For each clinical trial, 2 investigators from our team (including S.B., R.A.A.B., T.H., E.U., J.K.G., K.G., T.T., K.A., B.O., Y.C., D.-L.B., A.A., and S.G.) conducted title and abstract screening, full-text review, and data extraction using a standardized data extraction form that was incorporated into Covidence. A third investigator resolved disagreements via consensus in Covidence.

### Additional Source of Data: Uniform Data System

We retrieved FQHC characteristic information from the publicly available Uniform Data System (UDS) datasets from the Health Resources and Services Administration.^12^ Reporting to UDS is mandated for all FQHCs and look-alikes to submit their operational, patient characteristic, clinical, and outcome data on an annual basis. After identifying FQHCs engaged in the included clinical trials, we used the FQHC names to localize them and their unique identifier (4 digits in 2005-2010 databases and 10 digits in 2011-2023 databases) in the UDS database of the year in which the FQHC was initially engaged in the clinical trial (we approximated this to be the clinical trial start date). For studies that did not report the name of the FQHC engaged, we contacted the corresponding authors for that information. We retrieved FQHC organizational and patient demographic characteristics. Specifically, we retrieved data about FQHC location, annual patient volume, workforce, and electronic health record (EHR) capacity. Due to underreporting of workforce and EHR data before 2009, we excluded studies that began before that year. A graphic presentation of data sources and process of data acquisition is given as eFigure 1 in Supplement 1.

### Quality Appraisal

We used the National Heart, Lung, and Blood Institute of the National Institute of Health Quality Assessment Tool for controlled intervention studies to assess the quality of included articles.^13^ Two investigators scored each study independently; a third reviewed and resolved discrepancies (including S.B., R.A.A.B., T.H., E.U., J.K.G., K.G., T.T., K.A., B.O., Y.C., D.-L.B., A.A., and S.G.). Since our study included both clinical trial reports and protocols, we did not score protocols (9 of 14) or assign an overall rating score for these studies.^14,15,16,17,18,19,20,21,22^ Detailed strategy of quality assessment is presented in eMethods 2 and eTable 2 in Supplement 1.

### Synthesis Methods

To define the levels of FQHC engagement in clinical trials, we adapted the model of stakeholder engagement in research and the continuum of community engagement in research framework.^23,24^ We defined 4 levels of engagement, with higher levels indicating greater FQHC engagement (eFigure 2 in Supplement 1). Level 1 involved the FQHCs being informed but not participating in the design. Level 2 included FQHC consultation during the design phase and some engagement during implementation. Level 3 involved FQHCs as equal partners or initiators of the trial. Level 4 designated the FQHC as the lead in the project. To standardize the assignment of FQHC engagement levels, we developed 9 specific questions with responses of yes, no, and unclear. The questions are enumerated and the algorithm for this process is detailed in eMethods 3 in Supplement 1. Because of a small number of FQHCs engaged at levels 3 and 4, we combined these 2 levels during data analysis.

### Statistical Analysis

The analysis used FQHC data from the UDS to define 4 independent variables: location (urban or rural), patient volume (overall number of adult patients aged ≥18 years, and percentage of patients by age, sex, race, ethnicity, insurance status, and select diagnoses), workforce to patient ratio (full-time equivalents [FTEs] of clinicians typically involved in hypertension and T2D treatment and support staff to 10 000 adult patients), and EHR capacity (categorical variable). Propensity score weighting was applied to account for varying numbers of FQHCs engaged in each clinical trial, ensuring a balanced evaluation.^25^ FQHC characteristics were summarized using weighted means for patient volume and health workforce and frequency of facilities by location and EHR availability. Unadjusted and adjusted ordinal regression models assessed the association between FQHC characteristics and trial engagement levels. Results were presented as odds ratios (ORs) and 95% CIs, with significance defined at 2-sided *P* < .05. All analyses were performed in Stata/BE, version 17.0 (StataCorp LLC). Detailed data management and analysis methods are presented as eMethods 4 in Supplement 1.

## Results

### Search Results

Our systematic search for articles yielded 4552 references (Figure). We removed 1409 references from studies conducted before 2013, 1235 duplicates, and 1778 studies that did not meet inclusion criteria. Finally, we assessed the available full texts of 130 studies, which led to the exclusion of 97 studies, mostly because of missing full-text reports (n = 41) and the inability to match the reported FQHC in UDS to confirm that the FQHC was indeed an FQHC or a look-alike (n = 20). Overall, we included 33 articles in this review.^14,15,16,17,18,19,20,21,22,26,27,28,29,30,31,32,33,34,35,36,37,38,39,40,41,42,43,44,45,46,47,48,49^ Of the included references, 9 were study protocols lacking results of their corresponding RCTs,^14,15,16,17,18,19,20,21,22^ while 24 had results for their corresponding RCTs (Table 1).^26,27,28,29,30,31,32,33,34,35,36,37,38,39,40,41,42,43,44,45,46,47,48,49^ Eight studies included only populations with hypertension^18,19,20,22,32,43,44,45^; 22, populations with only T2D^14,16,17,21,26,27,28,29,30,31,34,35,36,37,38,39,40,41,42,46,48,49^; and 3, populations with both diseases.^15,33,47^ The geographical location of studies by US state and the number of FQHCs engaged is presented in eFigure 3 in Supplement 1.

**Figure.**
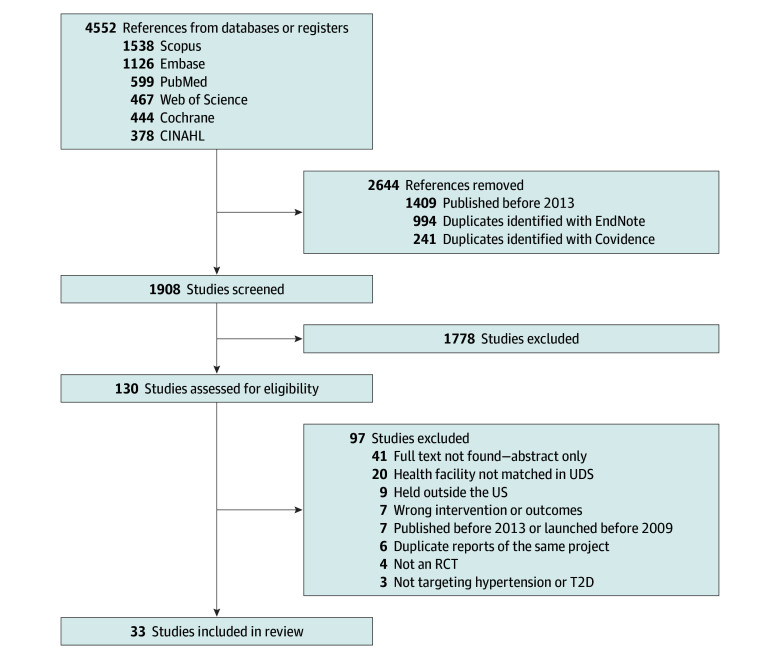
Study Flow Diagram CINAHL indicates Cumulative Index of Nursing and Allied Health Literature; RCT, randomized clinical trial; T2D, type 2 diabetes; UDS, uniform data system.

**Table 1.** Characteristics of Clinical Trials Included in the Systematic Review

Source (type)	Study location	Target population and disease focus	Purpose	RCT design	Facilities engaged, No. of sites/FQHC systems
Bluml et al,^27^ 2019 (report)	Chicago, Illinois	Adult patients (aged 21-85 y) with uncontrolled T2D	To test the efficacy of a telephonic diabetes support intervention to increase patient engagement in self-care using the health care system to improve clinical outcomes.	Parallel design	4/4
Bryce et al,^28^ 2021 (report)	Detroit, Michigan	Adult patients with uncontrolled T2D	To discern the impact of a fruit and vegetable prescription program compared with nonincentivized diabetes standard of care on changes in HbA_1c_ level, BP, and BMI.	Parallel design	1/1
Clark et al,^29^ 2020 (report)	San Diego and Riverside counties, California	Hispanic and low-income patients with uncontrolled T2D	To examine whether baseline levels of diabetes distress impacted clinical benefit from a mobile health DSME and support intervention.	Parallel design	NR/1
Commodore-Mensah et al,^22^ 2023 (protocol)	Different counties in Maryland	Patients aged 18-65 y with elevated untreated stage 1 HTN, chronic kidney condition, or history of a cardiovascular event	To compare the effect of the LINKED-BP program vs enhanced usual care on systolic BP reduction and use the RE-AIM framework to evaluate the reach, adoption, maintenance, and cost-effectiveness of the intervention at 12- and 24-mo post randomization.	Cluster RCT, effectiveness-implementation design	20/1
De Pue et al,^30^ 2013 (report)	Tafuna, American Samoa	Adult patients identifying as Samoan with T2D	To evaluate the effectiveness of a culturally adapted, primary care–based nurse-led CHW team intervention to support diabetes self-management on diabetes control and other biological measures.	Step-wedge design, cluster RCT	1/1
Delahanty et al,^31^ 2018 (report)	Eastern Massachusetts	Adult patients with T2D	To implement and test the effectiveness of an adapted Look AHEAD lifestyle intervention in clinical settings, including FQHC.	Parallel design	3/1
Deverts et al,^21^ 2022 (protocol)	Detroit, Michigan	Adult patients (aged 21-75 y) with uncontrolled T2D	To determine the effectiveness of Family Support for Health Action, a novel CHW-delivered DSME to patients and their support persons, relative to an established, CHW-delivered, individual patient–focused DSME and care management intervention.	Parallel design	1/1
Dodson et al,^20^ 2022 (protocol)	New York City, New York	Adults with uncontrolled HTN	To test whether a digitally enabled incentive lottery improves antihypertensive adherence and reduces systolic BP.	Parallel design	3/1
Fiscella et al,^32^ 2021 (report)	New York City, New York and New Jersey	Adult patients with uncontrolled HTN	To promote guideline adoption and assess follow-up time for patients with uncontrolled BP and systolic BP.	Step-wedge design, cluster RCT	12/10
Garrison et al,^33^ 2023 (report)	Missouri	Adult patients with uncontrolled HTN, T2D, or both	To test the efficacy of the Integrative Medication Self-Management Intervention in addressing medication adherence rates among community-dwelling adults with HTN, T2D, or both.	Pretest-posttest control group RCT	1/1
Hargraves et al,^19^ 2018 (protocol)	Lowell and Worcester, Massachusetts	Adults aged 30-75 y with uncontrolled HTN	To evaluate the implementation of a CHW-delivered culturally appropriate storytelling intervention for English- and Spanish-speaking patients diagnosed with HTN within FQHC.	Crossover design	2/2
Heisler et al,^36^ 2014 (report)	Detroit, Michigan	Adult patients with uncontrolled T2D	To compare outcomes between CHW use of a tailored, interactive web-based tablet-delivered tool (iDecide) vs use of print educational materials.	Parallel design	1/1
Heitkemper et al,^35^ 2017 (report)	New York City, New York	Adults with poor glycemic control (T2D)	To describe the characteristics and technology training needs of underserved adults with T2D who participated in a health information technology DSME intervention.	Parallel design, cluster RCT	8/5
Hessler et al,^36^ 2022 (report)	San Francisco, California	Adult patients (T2D)	To compare an evidence-based SMS program with an enhanced version that adds a patient engagement protocol, to elicit and address unique patient-level challenges to improve diabetes outcomes in FQHC.	Parallel design, cluster RCT	10/4
Ibe et al,^18^ 2021 (protocol)	Washington, DC, and Baltimore, Maryland	African American or Black women aged 40-75 y (HTN)	To evaluate the effectiveness of the Prime-Time Sister Circles Program on improved BP control, health care utilization attributed to cardiovascular events, and health care costs.	Parallel design	2/2
NCT05173675,^17^ 2024 (protocol)	Austin, Texas	Adult patients with uncontrolled T2D	To determine if a program that delivers empathetic and relationship-oriented phone calls by a friendly caller can support diabetes self-management behaviors and reduce HbA_1c_ in patients with diabetes at an FQHC.	Parallel design	1/1
Khanna et al,^37^ 2014 (report)	Oakland, California	Spanish-speaking adults with uncontrolled T2D	To determine whether automated telephone nutrition support counseling could help patients improve glycemic control by duplicating a successful pilot from Mexico in a Spanish-speaking US population.	Parallel design	1/1
Koonce et al,^38^ 2015 (report)	Nashville, Tennessee	Low-income adult patients (T2D)	To determine whether diabetes educational materials tailored to their health literacy levels and learning styles would positively impact participants’ knowledge of their condition.	Parallel design	1/1
Lindberg et al,^39^ 2021 (report)	Hillsboro, Oregon	Spanish-speaking women with overweight and T2D (diagnosis or at risk)	To develop a diabetes risk-reduction intervention responsive to the cultural practices of the Hispanic population that could be implemented in clinical settings serving this population to reduce body weight and waist circumference and improve markers of glycemic control (fasting blood glucose and HbA_1c_ levels) and cardiovascular risk (serum lipid profile), diet, and physical activity.	Parallel design	1/1
Mitchell et al,^40^ 2023 (report)	Boston, Massachusetts	Women with uncontrolled T2D	To develop an immersive telemedicine platform, linking an interactive virtual worlds learning environment with videoconferencing software to overcome the common barriers to diabetes group-based care while maintaining clinical effectiveness at scale in T2D management.	Parallel design	1/1
Nelson et al,^42^ 2018 (report)	Nashville, Tennessee	Adult patients receiving T2D treatment	To evaluate the effects of mobile phone–based diabetes support interventions on self-care and HbA_1c_ among adults with T2D.	Factorial Design	16/2
Persell et al,^43^ 2018 (report)	Chicago, Illinois	Patients with uncontrolled HTN	To test the effect of EHR-based medication support and nurse-led medication therapy management on HTN and medication self-management.	Parallel design, cluster RCT	12/1
Philis-Tsimikas et al,^15^ 2022 (protocol)	San Diego, California	Low-income Hispanic adults (T2D and HTN)	To compare the effectiveness of Dulce Digital, Dulce Digital-Me-Automated, and Dulce Digital-Me-Telephonic text messaging in improving diabetes clinical management for 12 mo.	Parallel design, Cluster RCT	11/1
NCT05195138,^16^ 2024 (protocol)	Birmingham, Alabama	Adults with T2D and moderate-to-severe diabetes distress	To compare and assess feasibility and acceptability of mindfulness-based diabetes education with standard DSME in adults with T2D and elevated diabetes distress who receive care within safety-net health care systems.	Parallel design	2/1
Redmond et al,^14^ 2023 (protocol)	Kansas City, Missouri	African American or Black patients (T2D)	To examine the efficacy of the web-based program eDECIDE relative to traditional DECIDE in improving HbA_1c_ levels through increased adherence to glucose monitoring, compliance to diabetes-related medications, and patient-clinician communication in African American participants with uncontrolled diabetes.	Parallel design	2/1
Shapiro et al,^44^ 2019	Los Angeles, California	Adults with uncontrolled HTN	To test a patient-centered intervention combining financial incentives with intrinsic motivation tools, to improve HTN control among adults attending FQHCs.	Parallel design	2/1
Shikany et al,^45^ 2023 (report)	North Carolina and Alabama	African American patients with uncontrolled HTN	To describe strategies for recruitment and retention of primary care practices in the Southeastern Collaboration and facilitators of and barriers to clinical trial participation.	Parallel design, cluster RCT	69/13
NCT0204359,^41^ 2023 (report)	Elm City, Tarboro, and Wilson, North Carolina	Low-income participants with uncontrolled T2D	To determine the impact of 2-way SMS on glycemic control in adults with low income and poorly controlled T2D.	Parallel design	3/1
Spencer et al,^46^ 2018 (report)	Detroit, Michigan	Hispanic adults 21 y or older (T2D)	To evaluate effectiveness of a CHW DSME program followed by 2 different approaches to maintaining improvements in HbA_1c_ level and other clinical and patient-centered outcomes for 18 mo.	Parallel design	1/1
Steinberg et al,^47^ 2018 (report)	Piedmont, North Carolina	Patients aged 21-65 y in racial and ethnic minority groups (T2D and HTN)	To examine the effect of a behavioral weight loss intervention among low-income, medically vulnerable adults on changes in diet quality.	Parallel design	4/1
Thom et al,^48^ 2013 (report)	San Francisco, California	Adult patients with uncontrolled T2D	To assess the impact of individual peer coaching on glucose control among patients with poorly controlled T2D attending public clinics.	Parallel design	6/4
Van Name et al,^49^ 2016 (report)	New Haven, Connecticut	Women aged 18-65 y ≥1 diabetes risk factor (T2D)	To test whether a diabetes prevention program intervention modified for an FQHC setting would decrease weight and improve metabolic measures in Hispanic women with prediabetes.	Parallel design	1/1
Welch et al,^26^ 2015 (report)	Springfield, Massachusetts	Hispanic population (T2D)	To compare a comprehensive diabetes team care condition involving use of an internet-based “diabetes dashboard” with a usual diabetes team care condition that does not have access to the dashboard.	Parallel design	2/2

Abbreviations: BMI, body mass index; BP, blood pressure; CHW, community health worker; DECIDE, Decision-Making Education for Choices in Diabetes Everyday; DSME, diabetes self-management education; EHR, electronic health record; FQHC, federally qualified health center; HbA_1c_, glycosylated hemoglobin; HTN, hypertension; NR, not reported; RCT, randomized clinical trial; RE-AIM, Reach, Effectiveness, Adoption, Implementation, Maintenance; SMS, short message service; T2D, type 2 diabetes.

### Quality Appraisal

Among the 33 studies included, we rated the quality in 1 as good,^43^ 13 as fair,^26,28,29,30,31,32,33,34,40,42,45,47,49^ and 10 as poor.^27,35,36,37,38,39,41,44,46,48^ The common reason for a poor rating was the loss to follow-up in the overall cohort of participants or higher differential loss to follow-up within 1 arm of the study. Detailed quality appraisal results are presented in eTable 2 in Supplement 1.

### Levels of FQHC Engagement in Hypertension and T2D Clinical Trials

In most cases, FQHC engagement in clinical trials occurred at levels 1 and 2 of engagement: 15 of 33 studies (46%) engaged 19 FQHCs at level 1^14,16,17,20,22,26,27,29,33,38,40,42,43,44,47^ and 8 of 33 studies (24%) engaged 38 FQHCs at level 2^18,19,30,32,35,36,39,45^ (Table 2). In these studies, FQHCs were most likely to be engaged in participant recruitment through the sharing of their EHR data with researchers to identify potential participants and intervention delivery. In most of these cases, FQHCs were engaged at the time of recruitment but not in the study planning and design phase.

**Table 2.** Included Studies and Levels of Federally Qualified Community Health Centers Engagement in Hypertension and Diabetes Clinical Trials

Source	Study site	Evaluation question and response^a^									Level of engagement^b^	Comments
		1	2	3	4	5	6	7	8	9		
Bluml et al,^27^ 2019	Chicago, Illinois	No	No	No	No	Unclear	Unclear	Yes	Yes	Unclear	1	Limited information about the role that the FQHC played
Clark et al,^29^ 2020	San Diego and Riverside, California	No	No	No	No	Unclear	Unclear	Unclear	Unclear	Yes	1	The role that FQHC coinvestigator in research design is unclear
Commodore-Mensah et al,^22^ 2023	Different counties in Maryland	No	No	No	No	No	Unclear	Yes	No	No	1	FQHC engagement limited to allowing access to EHR data
Dodson et al,^20^ 2022	New York City, New York	Unclear	No	No	No	No	No	No	Yes	No	1	Limited information about FQHC role beyond allowing access to EHR data
Garrison et al,^33^ 2023	Missouri	No	Unclear	Unclear	Unclear	Unclear	Yes	Yes	Yes	Unclear	1	Limited information about the exact roles that the FQHC played
NCT05173675,^17^ 2024	Austin, Texas	No	No	No	Unclear	Unclear	Unclear	Unclear	Unclear	Unclear	1	Limited information about FQHC roles beyond allowing recruitment from the site
Koonce et al,^38^ 2015	Nashville, Tennessee	No	No	Unclear	Unclear	Unclear	Unclear	Yes	Yes	No	1	FQHC operated by the research institution yet its role in the design of the project is unclear
Mitchell et al,^40^ 2023	Boston, Massachusetts	No	No	No	No	No	No	Yes	Yes	No	1	Limited information about the role that the FQHC played beyond recruitment and support in intervention delivery
Nelson et al,^42^ 2018	Nashville, Tennessee	No	No	No	No	No	No	Yes	No	No	1	Limited information about the role that the FQHC played beyond recruitment support
Persell et al,^43^ 2018	Chicago, Illinois	No	Unclear	No	No	Unclear	Unclear	Yes	Yes	Yes	1	FQHC collaborators did not play a role in study design as described in the author contribution
NCT05195138,^16^ 2024	Birmingham, Alabama	No	Unclear	Unclear	Unclear	No	No	Yes	No	Unclear	1	Limited information about the role that the FQHC will play
Redmond et al,^14^ 2023	Kansas City, Missouri	No	Unclear	Unclear	Unclear	Unclear	Unclear	Yes	No	No	1	FQHC engaged in initial recruitment, but researchers later opted for other means of recruitment
Shapiro et al,^44^ 2019	Los Angeles, California	No	No	No	No	No	Yes	Unclear	No	No	1	Limited information about the role that the FQHC played beyond allowing access to the patient database
Steinberg et al,^47^ 2018	Piedmont, North Carolina	No	No	No	No	No	Unclear	Yes	Yes	Yes	1	Clinicians supported intervention delivery, recruitment of participants, and dissemination of findings
Welch et al,^26^ 2015	Springfield, Massachusetts	No	Unclear	No	Unclear	Unclear	Unclear	Yes	Yes	Unclear	1	Clinicians received training to support recruitment and intervention delivery
De Pue et al,^30^ 2013	Tafuna, American Samoa	No	No	No	Unclear	Yes	No	Yes	Yes	Yes	2	Research team trained FQHC staff on RCT and American Diabetes Association guidelines
Fiscella et al,^32^ 2021	New York City, New York and New Jersey	No	No	No	No	No	Unclear	No	Yes	Unclear	2	Collaborated with CDN through which FQHC recruitment took place
Hargraves et al,^19^ 2018	Lowell and Worcester, Massachusetts	No	No	No	Unclear	Yes	Yes	Yes	Yes	No	2	Received input from community partners representing FQHC prior to initiating the intervention delivered by CHWs
Heitkemper et al,^35^ 2017	New York City, New York	No	No	No	Unclear	Yes	Yes	Yes	Yes	Unclear	2	Collaborated with CDN through which FQHC recruitment took place
Hessler et al,^36^ 2022	San Francisco, California	No	Unclear	No	Unclear	Unclear	Unclear	Yes	Yes	No	2	Project developed by research teams, trained FQHC to recruit and implement the project by existing staff in existing conditions
Ibe et al,^18^ 2021	Washington, D.C. and Baltimore, Maryland	No	No	No	No	Yes	Unclear	Yes	Yes	Unclear	2	FQHC assisted in the identification of eligible participants and recruitment (mass mailing)
Lindberg et al,^39^ 2021	Hillsboro, Oregon	No	Unclear	No	Unclear	Yes	Yes	Yes	Yes	No	2	FQHC were involved in the design through board review, but not in dissemination of findings
Shikany et al,^45^2023	North Carolina and Alabama	No	No	No	Unclear	Unclear	Yes	Yes	Yes	No	2	Study team assisted FQHC collaborators in certification on human subjects’ research
Deverts et al,^21^ 2022	Detroit, Michigan	No	No	No	No	Yes	Unclear	Yes	Yes	Yes	3	Study protocol developed using CBPR approaches at all stages
Heisler et al,^34^ 2014	Detroit, Michigan	No	No	No	No	Yes	No	Yes	Yes	Yes	3	Developed and implemented using CBPR principles with the REACH Detroit Partnership
Khanna et al,^37^ 2014	Oakland, California	No	Unclear	Unclear	Unclear	Yes	Yes	Yes	Yes	Yes	3	The academic institutional review board and the FQHC quality assurance subcommittee jointly reviewed and approved this study
Philis-Tsimikas et al,^15^ 2022	San Diego, California	No	No	No	Unclear	Yes	Yes	Yes	Yes	Yes	3	FQHC informed the research through sitting on advisory board and was engaged throughout the conduct of the project
Spencer et al,^46^ 2018	Detroit, Michigan	No	No	No	Yes	Yes	Yes	Yes	Yes	Yes	3	Developed and implemented using CBPR principles with the REACH Detroit Partnership
Van Name et al,^49^2016	New Haven, Connecticut	No	Yes	Yes	Unclear	Yes	Yes	Yes	Yes	Yes	3	FQHC and academic investigators collaborated to develop and implement this study
Bryce et al,^28^ 2021	Detroit, Michigan	Yes	Yes	Yes	Yes	Yes	Yes	Yes	Yes	Yes	4	Primary investigator is affiliated with both the academic institution and the FQHC. Further the study used CBPR principles at all stages
Delahanty et al,^31^ 2018	Eastern Massachusetts, Massachusetts	Yes	Yes	Yes	Yes	Yes	Unclear	Yes	Yes	Yes	4	All teams worked together, reviewed all materials, confirmed their understanding and comfort level with project plans
NCT0204359,^41^ 2023	Elm City, Tarboro, and Wilson, North Carolina	Yes	Yes	Yes	Yes	Yes	Yes	Yes	Yes	Yes	4	The principal investigator is affiliated with the FQHC
Thom et al,^48^ 2013	San Francisco, California	Yes	Yes	Yes	Yes	Yes	Unclear	Yes	Yes	Yes	4	All coauthors for both the protocol and the report are affiliated with both the FQHC and academic institution

Abbreviations: CBPR, community-based participatory research; CDN, clinical directors network; CHW, community health worker; EHR, electronic health record; FQHC, federally qualified health centers; EHR, Electronic Health Record; RCT, randomized clinical trial; REACH, Racial & Ethnic Approaches to Community Health.

^a^ The questions are enumerated in eMethods 3 in Supplement 1.

^b^ The levels of engagement are explained in eMethods 3 in Supplement 1.

For a small number of studies, investigators implemented higher levels of engagement of FQHCs. Six of 33 studies (18%) engaged FQHCs at level 3,^15,21,34,37,46,49^ and 4 of 33 studies (12%) engaged FQHCs at level 4.^28,31,41,48^ Of note, these partnerships were 1:1 between research institutions and FQHCs. In these types of engagement, FQHC were regarded as equal partners or were the sole implementers or coimplementers of the projects. In other instances, researchers were affiliated with both the FQHC and a research institution.

### FQHC Characteristics and Levels of Engagement in Clinical Trials

The characteristics of FQHCs that participated in the included clinical trials are presented in Table 3. Most health facilities were urban (52 [78%]) and had EHR capabilities (60 [90%]). The annual patient volume varied considerably by health facilities (expressed by wide SEs), and included FQHCs served a diverse group of patients. Annually, a mean (SE) of 33 777 (5481) patients were served by FQHCs engaged in clinical trials, slightly more than one-half were female (mean [SE], 56.8% [1.6%]), the largest age group was 65 years and older (mean [SE], 24.9% [1.7%]), and almost one-half were Hispanic or Latino (mean [SE], 49.4% [4.2%]). FQHCs with higher levels of engagement had higher percentages of Black or African American patients (mean [SE], 41.6% [8.9%] for levels 3 and 4 vs 31.8% [4.6%] overall; *P* = .047), lower percentages of White patients (mean [SE], 53.5% [7.6%] for levels 3 and 4 vs 60.1% [4.8%] overall; *P* = .051), and lower percentages of patients with private health insurance (mean [SE], 6.0% [1.4%] for levels 3 and 4 vs 12.5% [1.5%] overall; *P* = .003).

**Table 3.** Characteristics of Federally Qualified Community Health Centers Engaged in Hypertension and Type 2 Diabetes Clinical Trials Between 2009 and 2023 by Levels of Engagement

Characteristic	Mean (SE)				*P* value
	Overall (n = 67)	Level of engagement^a^			
		1 (n = 19)	2 (n = 38)	3 and 4 (n = 10)	
No. of patients (aged ≥18 y) served annually	33 777 (5481)	42 969 (10 047)	31 717 (6799)	20 654 (5570)	.07
Patients (aged ≥18 y) served annually by age groups, %					
18-24 y	11.9 (0.6)	11.8 (0.6)	12.3 (0.7)	11.7 (1.8)	.95
25-34 y	19.0 (0.8)	19.2 (1.3)	18.4 (0.7)	19.0 (2.0)	.92
35-44 y	17.1 (0.6)	17.2 (1.0)	15.8 (0.8)	18.0 (1.0)	.66
45-54 y	15.4 (0.5)	14.4 (0.6)	15.6 (1.4)	16.6 (0.8)	.03
55-64 y	11.8 (0.4)	11.4 (0.6)	12.1 (0.6)	12.1 (0.9)	.53
≥65 y	24.9 (1.7)	25.9 (2.3)	25.8 (2.9)	22.5 (3.7)	.46
Patients (aged ≥18 y) served annually by select diagnoses, %					
Diabetes	15.9 (2.4)	9.9 (1.0)	25.6 (5.3)	18.1 (5.6)	.09
Heart disease	4.5 (0.9)	3.6 (1.0)	4.8 (0.9)	5.8 (2.5)	.40
Hypertension	22.2 (2.9)	16.7 (1.7)	33.0 (5.7)	23.7 (7.3)	.22
Female patients (aged ≥18 y) served annually, %	56.8 (1.6)	56.8 (2.6)	56.2 (1.6)	57.2 (3.3)	.94
Hispanic or Latino patients (aged ≥18 y) served annually, %	49.4 (4.2)	45.7 (6.7)	44.8 (7.2)	59.4 (6.1)	.18
Patients (aged ≥18 y) served annually by race, %					
American Indian, Native Hawaiian, and other Pacific Islander	1.7 (0.5)	2.0 (0.9)	2.2 (0.9)	0.9 (0.3)	.38
Asian American	3.6 (0.8)	2.9 (0.9)	6.5 (2.3)	2.1 (1.1)	.93
Black or African American	31.8 (4.6)	22.0 (4.8)	39.0 (9.7)	41.6 (8.9)	.047
White	60.1 (4.8)	71.0 (5.7)	47.8 (10.5)	53.5 (7.6)	.051
Multiple race	2.7 (0.7)	2.1 (0.8)	4.6 (2.0)	1.9 (1.0)	.92
Patients (aged ≥18 y) served annually by health insurance coverage, %					
Uninsured	44.0 (4.1)	45.2 (5.5)	30.0 (6.1)	54.0 (8.3)	.53
Medicaid	32.4 (3.1)	27.7 (3.0)	43.9 (6.5)	29.9 (6.7)	.58
Medicare	10.0 (1.0)	9.9 (1.5)	10.3 (1.4)	9.9 (2.2)	.99
Other public insurance	1.1 (0.4)	1.4 (0.7)	1.7 (1.1)	0.2 (0.1)	.15
Private insurance	12.5 (1.5)	15.7 (2.5)	14.1 (1.5)	6.0 (1.4)	.003
Ratio of health workforce FTEs per 10 000 adult patients					
Physicians	4.08 (0.38)	3.02 (0.48)	4.43 (0.50)	5.47 (0.77)	.009
Family physicians	2.56 (0.37)	1.84 (0.34)	2.56 (0.62)	3.71 (0.91)	.06
General practitioners	0.16 (0.11)	0.02 (0.02)	0.30 (0.26)	0.27 (0.29)	.33
Internists	1.36 (0.25)	1.16 (0.28)	1.58 (0.31)	1.48 (0.68)	.62
Advanced practice clinicians	4.10 (0.35)	3.70 (0.45)	4.16 (0.53)	4.68 (0.86)	.31
Nurse practitioners	3.07 (0.35)	2.95 (0.50)	2.82 (0.43)	3.48 (0.81)	.61
Physician assistants	1.03 (0.12)	0.75 (0.16)	1.34 (0.33)	1.20 (0.12)	.03
Management and support personnel	8.26 (0.85)	6.91 (0.65)	7.78 (1.34)	10.82 (2.24)	.10
Patient and community education specialists	2.96 (0.57)	1.69 (0.62)	1.57 (0.38)	6.17 (1.08)	.002
Outreach specialist	1.03 (0.17)	0.86 (0.25)	1.17 (0.30)	1.19 (0.34)	.42
FQHC location, No. (%)					
Rural	15 (22)	5 (26)	9 (24)	1 (10)	.53
Urban	52 (78)	14 (74)	29 (76)	9 (90)	
EHR capability, No. (%)					
EHR system available	60 (90)	17 (89)	36 (95)	7 (70)	.64
EHR system lacking	2 (3)	1 (5)	1 (3)	0	
Data missing	5 (7)	1 (5)	1 (3)	3 (30)	

Abbreviations: EHR, electronic health record; FQHC, federally qualified health centers; FTE, full-time equivalents.

^a^ The levels of engagement are explained in eMethods 3 in Supplement 1.

Across the board, FQHCs with higher levels of engagement had higher ratios of workforce FTEs to patients. The overall physicians had a mean (SE) ratio of FTEs per 10 000 patients of 4.08 (0.38), and the advanced practice clinicians had a mean (SE) ratio of FTEs per 10 000 patients of 4.10 (0.35). Further, the medical services, management, and support personnel had a combined mean (SE) ratio of FTEs per 10 000 patients of 8.26 (0.85), while the specialized professionals who provided patient education services had a mean (SE) ratio of FTEs per 10 000 patients of 2.96 (0.57), and outreach services had a mean (SE) ratio of FTEs per 10 000 patients of 1.03 (0.17).

In the univariate ordinal regression models, association with the levels of FQHC engagement in clinical trials was found only for the physician (OR, 1.57; 95% CI, 1.16-2.13), management and support personnel (OR, 1.14; 95% CI, 1.02-1.27), and community and patient education specialist (CPES) (OR, 1.45; 95% CI, 1.09-1.91) ratios of FTE to patient variables. In the fully adjusted ordinal regression model, only physicians and CPES FTE to patient ratio variables were associated with the level of FQHC engagement in clinical trials (Table 4). A 1-unit increase in the physician FTE to patient ratio was associated with 54% higher odds of a higher level of engagement in clinical trials (OR, 1.54; 95% CI, 1.06- 2.23). Additionally, a 1-unit increase in the CPES FTE to patient ratio was associated with 41% higher odds of a higher level of engagement in clinical trials (OR, 1.41; 95% CI, 1.03-1.94). The proportional odds assumption testing with Brant test (χ^2^ = 5.03; *P* = .66) and gologit2 command (χ^2^ = 7.34; *P* = .39) indicated that the assumption was not violated. We assessed and found no association between levels of FQHC engagement in clinical trials and the time of intervention initiation or quality assessment outcomes of the clinical trials (eFigure 4 and eTables 3 and 4 in Supplement 1).

**Table 4.** Weighted Results of Ordinal Regression Model Between FQHC Characteristics and Level of Engagement in Hypertension and Type 2 Diabetes Clinical Trials

FQHC characteristic	OR (95%CI)	
	Unadjusted	Adjusted^a^
Patients (aged ≥18 y) served annually	0.99 (.99-1.00)	NA
Ratio of health workforce FTEs to patients^b^		
Physicians	1.57 (1.16-2.13)^c^	1.54 (1.06-2.23)^d^
Advanced practice providers	1.17 (0.88-1.56)	NA
Management support personnel	1.14 (1.02-1.27)^d^	0.98 (0.86-1.11)
Community and patient education specialists	1.45 (1.09-1.91)^c^	1.41 (1.03-1.94)^d^
Outreach specialists	1.20 (0.80-1.80)	NA
FQHC location, urban vs rural	2.54 (0.31-20.76)	NA

Abbreviations: FQHC, federally qualified health centers; FTE, full-time equivalents; NA, not applicable; OR, odds ratio.

^a^ Adjusted for disease type, FQHC location, and significant variables (*P* < .05) from unadjusted models.

^b^ Indicates odds of higher level of engagement associated with every 1-unit increase in FTE per 10 000 adult patients served annually.

^c^ *P* ≤ .01.

^d^ *P* < .05.

## Discussion

This systematic review provides a comprehensive overview of the current state of research on engagement in hypertension and T2D clinical trials in the US. We found limited literature on hypertension and T2D clinical trials involving FQHCs. Most clinical trials that engaged FQHCs did so at a limited level of engagement (level 1). Additionally, we found that an increase in physician and CPES FTEs is associated with higher levels of FQHC engagement in clinical trials. To our knowledge, our study is the first systematic review to explore the levels of FQHC engagement in clinical trials in the US and provide findings that are useful to researchers and other organizations looking to diversify their clinical trials by collaborating with FQHCs.

Given that hypertension and T2D are prevalent chronic conditions requiring ongoing management within health care settings, the low number of clinical trials involving FQHCs identified in this review is noteworthy. These findings partly explain the challenges that FQHCs face preventing their participation in clinical trials.^50^ A recent study that assessed barriers to clinical trial implementation for FQHCs further reported limited infrastructure, funding, and staffing.^51^ As noted, the low collaboration with FQHC in clinical trials on hypertension and T2D is a disservice to underserved populations who receive care in those health facilities and do not get an opportunity to learn about and engage in clinical trials.

We noted that higher physician and CPES FTE to patient ratios are associated with higher engagement of FQHCs in clinical trials for hypertension and T2D. This observation is consistent with previous reports, which suggest that FQHC prioritize clinical care due to often insufficient staff and resources allocated for research activities.^50,51^ Moreover, larger FQHC institutions, characterized by more complex organizational systems, may experience difficulties in engaging effectively in research, particularly in the absence of an established framework for research collaboration. Physicians play a critical role in assessing patients’ eligibility for research and recommending potential clinical trials that could be beneficial for them. CPES in FQHCs play a critical role in educating patients and the community, hence addressing the lack of awareness and knowledge about clinical trials.^52^ Whether more physicians or more health facilities lead this association is unclear; however, the general message remains the fact that higher resources in terms of workforce are needed for higher FQHC engagement in clinical trials.

Higher FQHC engagement in research could be beneficial in building FQHC research infrastructure, promoting diverse research participation, disseminating findings, and promoting health outcomes overall.^53,54^ However, we found that the FQHC influence on clinical trials is limited, and most researchers engage FQHCs to access data but do not contribute to building the infrastructure that produce those data. This is notable because, while FQHCs face critical resource and staff shortages, their engagement from the beginning could help identify how to address those challenges and integrate those needs in the study design and funding application. A clinical trial failed to introduce a mobile application for the self-management of hypertension and T2D within an FQHC because of multiple unforeseen obstacles.^55^ Clinicians reported a lack of time to educate and demonstrate the app to patients, and the lack of internet on site was another key obstacle to adoption.^55^ Such is an example of barriers researchers should be aware of while seeking to collaborate with FQHCs in research and address one project at a time until FQHCs are equipped to effectively engage in clinical research independently.

The lack of reliable funding for research forces FQHC to focus only on clinical care, often turning down research projects. In 2024, the National Institutes of Health announced that it would invest in testing the feasibility of integrating clinical research into community-based primary care settings such as FQHCs.^56^ For meaningful results, this funding will have to be used to build research capacity in primary care centers and set the stage for sustainable funding mechanisms for FQHC research. However, additional policies such as the establishment and funding of research offices at FQHCs and financial reimbursement of FQHCs for involvement in research activities will be needed.

### Limitations

Our study has some limitations. FQHC engagement in clinical trials is influenced by various factors beyond FQHCs themselves, including researchers, the type of research project, institutional policies, and funding opportunities, yet the present study did not explore those. Future research should investigate these additional factors affecting FQHC engagement. Another limitation is that our assessment of engagement levels depended on the information available in reports, which varied greatly due to factors like word count limits and insufficient journal avenues for describing stakeholder engagement in research. This limitation prevented us from exploring socioeconomic and cultural factors of clinical trial engagement by FQHC, and the possible heterogeneity across studies regarding levels of engagement. A well-designed qualitative study would be beneficial to explore the barriers and strategies for FQHC collaboration in clinical trials. Further, to advance our understanding of the role of health system and provider stakeholder engagement in research, researchers must provide greater detail on engagement methods. Additionally, 23 of 33 studies were rated as fair or poor quality, limiting the strength of the evidence, although we sought supplementary information to improve accuracy. Finally, the small number of FQHCs in our study limited subgroup analysis and prevented the exploration of how EHR availability affects levels of FQHC engagement in clinical trials.

## Conclusions

This systematic review evaluated FQHC engagement in hypertension and T2D clinical trials in the US and found limited research involving FQHCs. Most trials engaged FQHCs in the design phase, hence hindering their ability to build local research capacity. The present findings are important because greater FQHC involvement could help build trust with patients and promote diverse participation in clinical trials. Future studies should assess actionable steps for empowering FQHC toward research engagement and leading research studies.

## Notes

Supplement 1. **eMethods 1.** Article Search Summary **eMethods 2.** Quality Appraisal **eMethods 3.** Levels of Federally Qualified Community Health Center Engagement in Hypertension and Diabetes Clinical Trials **eMethods 4.** Data Management and Analysis **eTable 1.** Patient Intervention Comparison Outcome and Time **eTable 2.** Quality Assessment Using the Quality Assessment of Controlled Intervention Studies Criteria **eTable 3.** Weighted Levels of FQHC Engagement by the Clinical Trials’ Quality Assessment and Start Year **eTable 4.** Results of Weighted Ordinal Regression Models of Levels of FQHC Engagement and the Quality Assessment of the Clinical Trial and Intervention Start Year **eFigure 1.** Data Sources and Process for Data Acquisition **eFigure 2.** Levels of FQHC Engagement in Clinical Trials **eFigure 3.** Location of and FQHCs Engaged in Hypertension and Type 2 Diabetes Clinical Trials **eFigure 4.** Levels of FQHC Engagement in Clinical Trials Over Intervention Start Year
eReferences

Supplement 2.
Data Sharing Statement
