# Supplementary material for: Engagement in Hypertension and Diabetes Clinical Trials at Federally Qualified Health Centers: A Systematic Review
Source: JAMA Netw Open. 2025 Apr 15;8(4):e255258. doi: 10.1001/jamanetworkopen.2025.5258 (PMC12000987; doi:10.1001/jamanetworkopen.2025.5258)
Supplement: Supplement 1. — eMethods 1. Article Search Summary eMethods 2. Quality Appraisal eMethods 3. Levels of Federally Qualified Community Health Center Engagement in Hypertension and Diabetes Clinical Trials eMethods 4. Data Management and Analysis eTable 1. Patient Intervention Comparison Outcome and Time eTable 2. Quality Assessment Using the Quality Assessment of Controlled Intervention Studies Criteria eTable 3. Weighted Levels of FQHC Engagement by the Clinical Trials’ Quality Assessment and Start Year eTable 4. Results of Weighted Ordinal Regression Models of Levels of FQHC Engagement and the Quality Assessment of the Clinical Trial and Intervention Start Year eFigure 1. Data Sources and Process for Data Acquisition eFigure 2. Levels of FQHC Engagement in Clinical Trials eFigure 3. Location of and FQHCs Engaged in Hypertension and Type 2 Diabetes Clinical Trials eFigure 4. Levels of FQHC Engagement in Clinical Trials Over Intervention Start Year eReferences [file jamanetwopen-e255258-s001.pdf]

Supplementary Online Content

Byiringiro S, Barshja RAA, Hinneh T, et al. Engagement in hypertension and diabetes clinical trials at federally qualified health centers: a systematic review. *JAMA Netw Open.* 2025;8(4):e255258. doi:10.1001/jamanetworkopen.2025.5258

eMethods 1. Article Search Summary

eMethods 2. Quality Appraisal

eMethods 3. Levels of Federally Qualified Community Health Center Engagement in Hypertension and Diabetes Clinical Trials

eMethods 4. Data Management and Analysis

eTable 1. Patient Intervention Comparison Outcome and Time

eTable 2. Quality Assessment Using the Quality Assessment of Controlled Intervention Studies Criteria

eTable 3. Weighted Levels of FQHC Engagement by the Clinical Trials' Quality Assessment and Start Year

eTable 4. Results of Weighted Ordinal Regression Models of Levels of FQHC Engagement and the Quality Assessment of the Clinical Trial and Intervention Start Year

eFigure 1. Data Sources and Process for Data Acquisition

eFigure 2. Levels of FQHC Engagement in Clinical Trials

eFigure 3. Location of and FQHCs Engaged in Hypertension and Type 2 Diabetes Clinical Trials

eFigure 4. Levels of FQHC Engagement in Clinical Trials Over Intervention Start Year

eReferences.

This supplementary material has been provided by the authors to give readers additional information about their work.

© 2025 Byiringiro S et al. *JAMA Network Open*.

eMethods 1. Article Search Summary

**Topic**	FQHCs and Hypertension & T2 Diabetes
**Reference Manager**	EndNote, Covidence
**Databases**	PubMed, CINAHL, Embase, Cochrane, Web of Science, Scopus
**Date Run**	9/28, 10/2/2023
**Total Number of Results**	4552
**Search Prepared By**	Stella M. Seal, MLS
**Imported 4552 into EndNote (Sam – Nov 6, 2023)**	**Removed those conducted before 2013 (#1409)** Remaining: **3143**
**EndNote by Sam (Sam – Nov 6, 2023)**	Removed Duplicates (#**994**) Remaining: **2,149**
**2149 Imported to Covidence (Sam – Nov 6, 2023)**	Removed Duplicates: **241** Remaining for Title and Abstract review: 1908

PubMed

Search number	Query	Results
5	#1 AND #2 AND (#3 OR #4)	599
4	"Randomized Controlled Trials as Topic"[Mesh]	168,002
3	((randomized controlled trial[pt]) OR (controlled clinical trial[pt]) OR (randomized[tiab] OR randomised[tiab]) OR (placebo[tiab]) OR (drug therapy[sh]) OR (randomly[tiab]) OR (trial[tiab]) OR (groups[tiab]))	5,865,459
2	("Hypertension"[Mesh]) OR "Diabetes Mellitus, Type 2"[Mesh] OR hypertens* [tiab] OR "high blood pressure" [tiab:~3] OR "elevated blood pressure" [tiab:~3] OR diabet* [tiab] OR NIDDM [tiab] OR MODY [tiab]	1,281,031
1	"Community Health Centers"[Mesh] OR "community health center" [tiab:~3] OR "community health centers" [tiab:~3] OR "fqchc" [tiab] OR "neighborhood health center" [tiab:~3] OR "federally qualified center" [tiab:~2] OR "neighborhood health centers" [tiab:~2] OR "neighborhood health clinic" [tiab:~3] OR "neighborhood health clinics" [tiab:~3] OR "tribal health center*" [tiab] OR "tribal health clinic*" [tiab] OR "ihs clinic" [tiab] OR "migrant health center*" [tiab] OR "migrant health clinic*" [tiab] OR "healthcare for the homeless" [tiab] OR "nurse managed health clinic*" [tiab] OR nmhc* [tiab] OR "nurse managed health center*" [tiab] OR "public health clinic*" [tiab] OR "urban health center*" [tiab] OR "rural health center*" [tiab] OR "urban health clinic*" [TIAB] OR "rural health clinic*" [tiab]	22,066

Embase

No.	Query	Results
#4	#1 AND #2 AND #3	1126

© 2025 Byiringiro S et al. *JAMA Network Open*.

#3	'crossover procedure'/de OR 'double-blind procedure'/de OR 'randomized controlled trial'/de OR 'single-blind procedure'/de OR random*:de,ab,ti OR factorial*:de,ab,ti OR crossover*:de,ab,ti OR ((cross NEXT/1 over*):de,ab,ti) OR placebo*:de,ab,ti OR ((doubl* NEAR/1 blind*):de,ab,ti) OR ((singl* NEAR/1 blind*):de,ab,ti) OR assign*:de,ab,ti OR allocat*:de,ab,ti OR volunteer*:de,ab,ti	3226682
#2	'non insulin dependent diabetes mellitus'/exp OR 'hypertension'/exp OR 'hypertension':ti,ab,kw OR 'diabetes mellitus, type 2':ti,ab,kw OR hypertens*:ti,ab,kw OR (((high OR elevated) NEAR/3 'blood pressure'):ti,ab,kw) OR diabet*:ti,ab,kw OR niddm:ti,ab,kw OR mody:ti,ab,kw	2316813
#1	'health center'/exp OR 'community health centers':ti,ab,kw OR (((community OR neighborhood OR tribal OR migrant OR 'nurse managed' OR urban OR public OR rural OR 'federally qualified') NEAR/3 ('health center*' OR 'health clinic*')):ti,ab,kw) OR 'fqchc':ti,ab,kw OR 'healthcare for the homeless':ti,ab,kw OR 'ihs clinic':ti,ab,kw OR nmhc*:ti,ab,kw	56417

CINAHL

#	Query	Limiters/Expanders	Last Run Via	Results
S5	S1 AND S2 AND (S3 OR S4)	Expanders - Apply related words; Apply equivalent subjects Search modes - Boolean/Phrase	Interface - EBSCOhost Research Databases Search Screen - Advanced Search Database - CINAHL Plus with Full Text	378
S4	(MH "Randomized Controlled Trials+")	Expanders - Apply related words; Apply equivalent subjects Search modes - Boolean/Phrase	Interface - EBSCOhost Research Databases Search Screen - Advanced Search Database - CINAHL Plus with Full Text	139,532
S3	(randomized controlled trials OR MH double-blind studies OR MH single-blind studies OR MH random assignment OR MH pretest-posttest design OR MH cluster sample OR TI (randomised OR randomized) OR AB (random*) OR TI (trial) OR (MH (sample size) AND AB (assigned OR allocated OR control)) OR MH (placebos) OR PT (randomized controlled trial) OR AB (control W5 group) OR MH (crossover design) OR MH (comparative studies) OR AB (cluster W3 RCT))	Expanders - Apply related words; Apply equivalent subjects Search modes - Boolean/Phrase	Interface - EBSCOhost Research Databases Search Screen - Advanced Search Database - CINAHL Plus with Full Text	1,029,167
S2	((MH "Hypertension+") OR (MH "Diabetes Mellitus, Type 2")) OR TI ("Hypertension" OR "Diabetes Mellitus, Type 2" OR hypertens* OR ((high OR	Expanders - Apply related words; Apply equivalent subjects	Interface - EBSCOhost Research Databases Search Screen - Advanced Search	373,120

© 2025 Byiringiro S et al. *JAMA Network Open*.

	elevated) N3 “blood pressure") OR diabet* OR NIDDM OR MODY) OR AB ("Hypertension" OR "Diabetes Mellitus, Type 2” OR hypertens* OR ((high OR elevated) N3 “blood pressure") OR diabet* OR NIDDM OR MODY)	Search modes - Boolean/Phrase	Database - CINAHL Plus with Full Text	
S1	((MH "Community Health Centers") OR (MH "Rural Health Centers")) OR TI ("Community Health Centers" OR ((community OR neighborhood OR tribal OR migrant OR “nurse managed” OR urban OR public OR rural OR “federally qualified”) N3 (“health center*” OR “health clinic*”)) OR “fqchc” OR “healthcare for the homeless” OR “ihs clinic” OR nmhc*) OR AB ("Community Health Centers" OR ((community OR neighborhood OR tribal OR migrant OR “nurse managed” OR urban OR public OR rural OR “federally qualified”) N3 (“health center*” OR “health clinic*”)) OR “fqchc” OR “healthcare for the homeless” OR “ihs clinic” OR nmhc*)	Expanders - Apply related words; Apply equivalent subjects Search modes - Boolean/Phrase	Interface - EBSCOhost Research Databases Search Screen - Advanced Search Database - CINAHL Plus with Full Text	13,902

Cochrane

Search Name:
Date Run: 02/10/2023 11:19:28

ID	Search	Hits
#1	MeSH descriptor: [Community Health Centers] explode all trees	670
#2	"Community Health Centers" OR ((community OR neighborhood OR tribal OR migrant OR “nurse managed” OR urban OR public OR rural OR “federally qualified”) NEAR/3 (“health center*” OR “health clinic*”)) OR “fqchc” OR “healthcare for the homeless” OR “ihs clinic” OR nmhc*	1873
#3	#1 OR #2	2277
#4	MeSH descriptor: [Hypertension] explode all trees	26651
#5	MeSH descriptor: [Diabetes Mellitus, Type 2] explode all trees	23411
#6	"Hypertension" OR "Type 2 Diabetes Mellitus” OR hypertens* OR ((high OR elevated) NEAR/3 “blood pressure") OR diabet* OR NIDDM OR MODY	186514
#7	#4 OR #5 OR #6	186514
#8	#3 AND #7	444

Web of Science

(TS=("Community Health Centers" OR ((community OR neighborhood OR tribal OR migrant OR “nurse managed” OR urban OR public OR rural OR “federally qualified”) NEAR/3 (“health center*” OR “health clinic*”)) OR “fqchc” OR “healthcare for the homeless” OR “ihs clinic” OR nmhc*)) AND TS=("Hypertension" OR "Type 2 Diabetes Mellitus” OR hypertens* OR ((high OR elevated) NEAR/3 “blood pressure") OR diabet* OR NIDDM OR MODY) AND TS=((random* OR

© 2025 Byiringiro S et al. *JAMA Network Open*.

sham OR placebo*) OR ((singl* OR doubl*) NEAR/1 (blind* OR dumm* OR mask*)) OR ((tripl* OR trebl*) NEAR/1 (blind* OR dumm* OR mask*)) OR (control* NEAR/3 (study OR studies OR trial* OR group*)) OR (clinical NEAR/3 (study OR studies OR trial*)) OR (Nonrandom* OR "non random*" OR non-random* OR quasi-random* OR quasirandom*) OR (phase NEAR/3 (study OR studies OR trial*)) OR ((crossover OR cross-over) NEAR/3 (study OR studies OR trial*)) OR ((multicent* OR multi-cent*) NEAR/3 (study OR studies OR trial*)) OR (allocated) OR (("open label" OR open-label) NEAR/5 (study OR studies OR trial*)) OR ((equivalence OR superiority OR non-inferiority OR noninferiority) NEAR/3 (study OR studies OR trial*)) OR ("pragmatic study" OR "pragmatic studies") OR ((pragmatic OR practical) NEAR/3 trial*) OR ((quasiexperimental OR quasi-experimental) NEAR/3 (study OR studies OR trial*)) OR (trial) OR (trial))

Searched 10/2/23

467 results

Scopus

TITLE-ABS-KEY("Community Health Centers" OR (community W/3 "health center*") OR (community W/3 "health clinic*") OR (neighborhood W/3 "health center*") OR (neighborhood W/3 "health clinic*") OR (Tribal W/3 "health center*") OR (tribal W/3 "health clinic*") OR (migrant W/3 "health center*") OR (migrant W/3 "health clinic*") OR ("nurse managed" W/3 "health center*") OR ("nurse managed" W/3 "health clinic*") OR (urban W/3 "health center*") OR (urban W/3 "health clinic*") OR (public W/3 "health center*") OR (public W/3 "health clinic*") OR (rural W/3 "health center*") OR (rural W/3 "health clinic*") OR ("federally qualified" W/3 "health center*") OR ("federally qualified" W/3 "health clinic*") OR "fqchc" OR "healthcare for the homeless" OR "ihs clinic" OR nmhc) AND TITLE-ABS-KEY("Hypertension" OR "Type 2 Diabetes Mellitus" OR hypertens* OR (high W/3 "blood pressure") OR (elevated W/3 "blood pressure") OR diabet* OR NIDDM OR MODY) AND (TITLE-ABS-KEY (random* OR sham OR placebo*) OR TITLE-ABS-KEY ((singl* OR doubl*) W/1 (blind* OR dumm* OR mask*)) OR TITLE-ABS-KEY ((tripl* OR trebl*) W/1 (blind* OR dumm* OR mask*)) OR TITLE-ABS-KEY (control* W/3 (study OR studies OR trial* OR group*)) OR TITLE-ABS-KEY (clinical W/3 (study OR studies OR trial*)) OR TITLE-ABS-KEY (Nonrandom* OR "non random*" OR non-random* OR quasi-random* OR quasirandom*) OR TITLE-ABS-KEY (phase W/3 (study OR studies OR trial*)) OR TITLE-ABS-KEY ((crossover OR cross-over) W/3 (study OR studies OR trial*)) OR TITLE-ABS-KEY ((multicent* OR multi-cent*) W/3 (study OR studies OR trial*)) OR TITLE-ABS (allocated) OR TITLE-ABS-KEY (("open label" OR open-label) W/5 (study OR studies OR trial*)) OR TITLE-ABS-KEY ((equivalence OR superiority OR non-inferiority OR noninferiority) W/3 (study OR studies OR trial*)) OR TITLE-ABS-KEY ("pragmatic study" OR "pragmatic studies") OR TITLE-ABS-KEY ((pragmatic OR practical) W/3 trial*) OR TITLE-ABS-KEY ((quasiexperimental OR quasi-experimental) W/3 (study OR studies OR trial*)) OR TITLE (trial) OR KEY (trial))

Searched 10/2/23

1538 ords

© 2025 Byiringiro S et al. *JAMA Network Open*.

eMethods 2. Quality Appraisal

We used the National Heart, Lung, and Blood Institute of the National Institute of Health Quality Assessment Tool for controlled intervention studies to assess the quality of included articles.[1] This tool assesses 14 key elements of the study design and implementation to assign an overall quality rating as "good," "fair," or "poor." Studies with "poor" overall quality ratings were those judged to have low internal validity, as evidenced by the presence of at least one "fatal flaw." Fatal flaws in the design or implementation of the clinical trial were instances that could significantly increase the risk of bias, including high attrition rates, high differential dropout rates, and lack of intention-to-treat analysis or unsuitable statistical analysis. We rated a study to have "fair" quality if it did not meet all 14 aspects of the criteria but had no fatal flaw. We rated a study as "good" if it met all of the 14 criteria of the assessment tool. Two investigators scored each study independently; a third reviewed and resolved discrepancies. Since our study included both clinical trial reports and protocols, we did not score protocols (9 out of 14) or assign an overall rating score for these studies.

© 2025 Byiringiro S et al. *JAMA Network Open*.

eMethods 3. Levels of Federally Qualified Community Health Center Engagement in Hypertension and Diabetes Clinical Trials

Answer the following questions from your assessment of the study report and/or protocol and any other provided study documents (Yes/No/Unclear)

(1) Is the PI from the FQHC

(2) Did FQHC conceive the research idea and sought support for the research?

(3) Did FQHC fund the research or seek funding by itself?

(4) Were FQHC engaged in funding acquisition?

(5) Were FQHC engaged in the design of the study/planning phase?

(6) Did FQHC personnel sit on the trial's advisory board?

(7) Were FQHC engaged in the recruitment phase?

(8) Were FQHC engaged in the intervention delivery?

(9) Did at least one FQHC personnel participate in manuscript writing/publication (co-authorship)?

What is your judgement of the overall level of FQHC engagement [single choice]?

Informed by the Continuum of Community Engagement in Research.[2]

- Level 1 - FQHC informed.
- Level 2 - FQHC consultation and/or participation
- Level 3 - FQHC initiated or as an equal partner.
- Level 4 - FQHC driven project.

Instructions for assigning a level of FQHC engagement

- ***Level 1 -*** *Absence of FQHC co-author OR Presence of FQHC co-author who only supported implementation but not the design OR Lack of description of the role played by FQHC in Study design.* ***Check methods and author contributions section.*** **[#1-6 are NO or Unclear]**

© 2025 Byiringiro S et al. *JAMA Network Open*.

Case Example 1: A clinical trial is testing a blood pressure control program targeting Black or African American and Latino populations. The study initially aims to recruit participants from the general community using a variety of recruitment methods. However, during implementation, researchers encounter challenges in meeting recruitment goals. To address this, they partner with FQHCs to recruit participants. The FQHC staff collaborate with the researchers, helping them achieve their recruitment targets. Following participant recruitment, the intervention is successfully delivered, and data analysis is completed. The researchers then publish their findings and formally acknowledge the support provided by the FQHC.

- ***Level 2*** *- Researcher sought and received FQHC feedback during the design of the study.*

  FQHC personnel may hold a defined role in the study (recruitment, community advisory boards, study chairmanship…). ***Check methods and author contribution if FQHC co-author contributed to the design, implementation and reporting of the study. [#5, OR 6 are YES AND #7, 8 or 9 are YES].***

Case Example 2: A pilot trial of a diabetes self-management intervention is seeking to recruit participants from various primary care clinics (FQHC and not). They are planning to engage some FQHC in their geographic location and during the design of the study, they reach out to FQHC to seek feedback about the intervention itself, methods of recruitment, and planned dissemination. FQHCs provide extensive feedback some of which the researchers decide to implement. Some FQHCs accept to be part of the study, provided the project does not take much of their time. Some clinical staff at the FQHCs join the advisory board of the trial. The trial is implemented successfully and the champions at the partnering FQHCs participate in the dissemination of findings.

- ***Level 3*** *- FQHC personnel conceived the research idea. OR Project co-developed with FQHC personnel OR*

  FQHC leadership sought partnership to help answer research question AND FQHC participates in all phases of the research continuum from ideation to dissemination. ***[#2, 5, 7, 8, 9 are YES] or [#4-9 are YES]***

Case Example 3: A clinical trial is seeking to test a medication adherence intervention among patients co-morbid with hypertension and T2DM who receive care at FQHCs. The study reaches a memorandum of understanding with three FQHC each of which as a representative member. The FQHCs participate in the design of the project and funding

© 2025 Byiringiro S et al. *JAMA Network Open*.

acquisition process. In this stage, FQHC get an opportunity to include potential expenses related to equipping their labs, additional needs in staff and their training on research and the intervention, internet infrastructure for the staff who will partner on this project. The agreement requires the clinical trial to have an academic and FQHC co-PI and they have to have equal voting powers. The FQHCs are involved through the whole steps of the clinal trial and will benefit from any benefits that could result from the trial.

- ***Level 4 -*** *Project led by a FQHC PI with or without academic or non-academic research partner.*

  FQHC PI sought funding or FQHC funded the research. ***[#1 is YES] OR [#1 AND 3 AND 5-9 are YES]***

Case Example 4: A FQHC staff member is interested in testing a successful quality improvement project for hypertension management in a pilot clinical trial. She recruits patients and fellow clinicians at his and nearby FQHC. She carefully designs a project, seeks funding, and implements the project from initiation to completion.

© 2025 Byiringiro S et al. *JAMA Network Open*.

eMethods 4. Data Management and Analysis

eMethods 4.1. Data management

To assess the FQHC characteristics associated with levels of engagement in clinical trials, we retrieved four variables from UDS: location, patient volume, workforce, and EHR capacity. FQHC location was coded as a binary variable (urban/rural). The variable about the patient volume in UDS is presented as the number of patients served annually (a continuous variable). Since hypertension and T2DM are prevalent among adults, we retrieved the number of patients ($\geq$18 years) treated at each FQHC annually. While we did not include these in the association analyses because of small sample size, we characterized the overall number of adult patients served by FQHCs per year and the percentage of adult patients by age categories (18 to 24, 25 to 34, 35 to 44, 45 to 54, 55 to 64, and 65 and above), biological sex (female), ethnicity (Hispanic), race (African American; White; Asian American; American Indian, Native Hawaiian, and other Pacific Islanders; and mixed race), health insurance coverage status (uninsured, Medicaid, Medicare, Other Public Insurance, and Private Insurance), and select diagnoses (hypertension, and diabetes).

We included the full-time equivalents (FTE) (as a continuous variable) of five health workforce categories at the FQHC that participated in the included clinical trials. For clinicians, we included those involved closely in managing patients with hypertension and T2DM. These were physicians (family physicians, general practitioners, and internists), advanced practice providers (nurse practitioners and physician assistants), management and support personnel, community and patient education specialists (CPES), and outreach specialists. For ease of comparison across levels of FQHC engagement in clinical trials, we indexed the FTE of health workforce to the number of adult patients served annually. To calculate the indexed health workforce FTE, we estimated the number of FTE needed to serve 10,000 adult patients annually. Here is the formula we followed:

$$Health\ workforce\ FTE\ to\ Patient\ Ratio\ = \frac{FTE\ of\ health\ workforce(e.g. physicians)}{Patients(\geq 18\ yrs)\ served\ anually} \times 10{,}000$$

© 2025 Byiringiro S et al. *JAMA Network Open*.

The EHR capacity was a binary categorical variable. Despite the different types of EHR systems with varying functionalities, UDS does not provide this specification. EHR availability variable data was missing for some FQHCs.

The outcome of interest was the level of FQHC engagement in hypertension and T2DM clinical trials. Given that studies engaged different numbers of FQHCs, and the outcome of interest was defined at the study level, we sought to apply weights to avoid the over-inflation of the effect of the outcome of a study that engaged many compared to studies that engaged a single or smaller number of FQHCs. We used propensity score weighting to define weights.[3] Each FQHC was assigned a weight equivalent to the inverse proportion of the number of FQHCs engaged and identified from UDS. For instance, if a study engaged four FQHCs that we identified from UDS, the weight of each FQHC engaged by that study was a quarter (¼), and if another study engaged a single FQHC, the weight of that FQHC would be one (1). Additionally, since the number of FQHCs engaged at level 4 was too small (only 4 FQHCs), we combined levels 3 and 4 while exploring the FQHC characteristics associated with higher levels of engagement in clinical trials.

eMethods 4.2. Analysis

We summarized the characteristics of FQHCs engaged in clinical trials by the corresponding level of engagement. We used weighted mean and standard error (SE) of the patient volume and stratified the patient volume by age categories, biological sex, race, ethnicity, health insurance coverage, and specific diagnoses (hypertension and T2DM). Further, we presented the mean (SE) full-time equivalents of the health workforce stratified by the FQHCs' level of engagement in clinical trials. We used weighted analysis of variance to compare continuous variables by level of engagement of FQH in clinical trial. We summarized the frequency and proportion of health facilities by location and EHR availability and used chi-square test to compare these variables by the level of FQHC engagement in clinical trials.

We conducted unadjusted and adjusted ordinal regression models to assess the association between FQHC characteristics and levels of FQHC engagement in hypertension and T2DM clinical trials. The outcome of interest was the level of FQHC engagement as an ordinal variable. Initially, we conducted weighted univariate ordinal regression models between each FQHC characteristic (patient volume, the health workforce FTE to patient ratio variables, and the FQHC location) and the level of FQHC engagement. Only variables with significant association with the outcome ($p<0.05$) in the univariate models

© 2025 Byiringiro S et al. *JAMA Network Open*.

were included in the final (adjusted) model. The final model was further adjusted for the disease type (hypertension, T2DM, or both), and location of the FQHC. Since almost all FQHCs had EHR systems available, we did not assess its association with levels of FQHC engagement in clinical trials. Further, given a small number of FQHCs, we did not conduct sub-group analyses of patient volume by categories and health workforce types of physicians and advanced practice providers. We used brant test[4] and gologit2[5,6] commands to test for the violation of proportional odds assumption. Both these commands test the hypothesis that the proportional odds assumption is not violated. A likelihood ratio chi-square with a non-significant p-value (>0.05) leads to the failure to reject the null hypothesis and conclusion that the proportional odds assumption is not violated. We performed sensitivity analyses to explore the longitudinal association between intervention start date and the level of engagement in clinical trials, and the association between the quality of published studies and the level of engagement in clinical trials. The ordinal regression results were presented as odds ratios, 95% confidence interval (CI), and significant p-values. The significance level was set as p<0.05. We conducted all analyses in Stata/BE 17.0.

eTable 1. Patient Intervention Comparison Outcome and Time

PICOT	Operationalization
**Population/Patients**	• Adults aged ≥18 years and<br>• Diagnosed with hypertension or type 2 diabetes mellitus (T2DM)
**Interventions**	• All types randomized clinical trials conducted at or with FQHC or Look-Alikes to address hypertension or T2DM
**Comparison**	• Setting (Rural/urban)<br>• Patient volume<br>• Full-time equivalent of health workforce<br>• Health information capabilities (use of Electronic Health Record)
**Outcome**	• Levels of FQHC Engagement*
**Time**	• Published from 2013 until today

*Defined by adapting Continuum of Community Engagement in Research

Abbreviations: FQHC – Federally Qualified Health Center, T2DM – Type 2 Diabetes Melitus.

© 2025 Byiringiro S et al. *JAMA Network Open*.

eTable 2. Quality Assessment Using the Quality Assessment of Controlled Intervention Studies Criteria

Study	Q1	Q2	Q3	Q4	Q5	Q6	Q7	Q8	Q9	Q10	Q11	Q12	Q13	Q14	Overall Quality	Comments
Bluml et al, 2019[7]	√	√	–	×	×	√	√	×	–	–	√	×	√	×	Poor	Only 42% completed the study and no information whether study used intention to treat analysis.
Bryce et al, 2021[8]	√	√	×	×	×	√	√	√	√	√	√	–	×	√	Fair	
Clark et al, 2020[9]	√	√	√	×	×	√	√	√	√	√	√	×	√	√	Fair	
Commodore-Mensah et al, 2023[10]	√	√	–	–	√	–	–	–	–	–	–	–	–	–	NA	Protocol
Delahanty et al, 2018[11]	√	√	√	×	√	√	√	√	√	–	√	√	√	√	Fair	
De Pue et al, 2013[12]	√	√	–	×	×	√	√	√	√	√	√	×	√	√	Fair	
Deverts et al, 2022[13]	√	√	√	×	√	–	–	–	–	√	√	–	√	√	NA	Protocol
Dodson et al, 2022[14]	√	√	–	–	–	–	–	–	–	–	√	√	–	–	NA	Protocol
Fiscella et al, 2021[15]	√	√	–	×	×	√	√	√	–	–	√	√	√	√	Fair	
Garrison et al, 2023[16]	√	√	√	×	×	√	√	√	√	√	√	√	√	√	Fair	
Hargraves et al, 2018[17]	√	√	√	√	×	–	–	–	–	–	√	√	–	–	NA	Protocol
Heisler et al, 2014[18]	√	√	√	×	√	√	√	√	√	√	√	√	√	√	Fair	
Heitkemper et al, 2017[19]	√	–	–	–	–	–	–	–	–	√	√	–	–	–	Poor	Multiple components of the design are missing
Hessler et al, 2022[20]	√	√	√	–	×	√	×	√	–	–	√	√	√	√	Poor	25.5% were lost to follow-up in the enhanced intervention group
Ibe et al, 2021[21]	√	√	√	√	×	–	–	–	–	–	–	√	–	–	NA	Protocol
Kahlon et al, 2023[22]	√	–	–	–	√	–	–	–	–	–	–	–	–	–	NA	Protocol
Khanna et al, 2014[23]	√	√	√	–	√	√	×	×	×	–	√	√	√	√	Poor	Only 65% in the intervention arm completed the second and final visits.
Koonce et al, 2015[24]	√	√	×	–	×	√	×	√	–	×	√	√	√	√	Poor	25.7% loss to follow-up in the intervention arm at 6 weeks
Lindberg et al, 2021[25]	√	√	–	×	√	√	√	–	–	–	√	√	√	–	Poor	Not clear if ITT was applied. 28.7% overall drop-out at 12 months.
Mitchell et al, 2023[26]	√	√	√	×	×	√	√	√	√	√	√	√	√	√	Fair	

© 2025 Byiringiro S et al. *JAMA Network Open*.

Nelson et al, 2018[27]	√	√	√	×	–	√	√	√	√	×	√	√	√	√	Fair	
Persell et al, 2018[28]	√	√	√	√	√	√	√	√	√	√	√	√	√	√	Good	
Philis-Tsimikas et al, 2022[29]	√	–	–	–	–	–	–	–	–	–	–	–	–	–	NA	Protocol
Presley et al, 2023[30]	√	–	–	√	–	–	–	–	–	–	–	–	–	–	NA	Protocol
Redmond et al, 2023[31]	√	√	–	–		–	–	–	–	√	√	×	√	–	NA	Protocol
Shapiro et al, 2019[32]	√	√	√	×	×	×	×	√	√	√	√	√	√	√	Poor	24.3% and 21.1% loss to follow-up in the intervention and control respectively
Shikany et al, 2023[33]	√	√	√	×	×	√	√	√	√	–	√	√	×	√	Fair	
Smith et al, 2023[34]	√	√	–	×	×	√	×	×	–	–	√	×	√	–	Poor	27.4% and 25.3% loss to follow-up in control and intervention groups respectively.
Spencer et al, 2018[35]	√	√	√	×	–	×	×	×	–	–	√	×	√	√	Poor	Overall dropout rate higher than 20% at 18 months
Steinberg et al, 2018 [36]	√	√	√	×	–	√	√	–	–	–	√	√	–	√	Fair	
Thom et al, 2013[37]	√	√	√	×	×	–	√	×	–	–	√	√	√	√	Poor	50% higher drop out in the usual than health coaching arms.
Van Name et al, 2016[38]	√	√	–	–	–	×	√	√	√	–	√	√	√	√	Fair	
Welch et al, 2015[39]	√	√	–	–	–	√	√	√	–	–	√	×	√	√	Fair	

Yes (√), No (×), Other-CD, NR, NA (–)

Criteria (Yes/No/Other (CD, NR, NA))

Q1. Was the study described as randomized, a randomized trial, a randomized clinical trial, or an RCT?
Q2. Was the method of randomization adequate (i.e., use of randomly generated assignment)?
Q3. Was the treatment allocation concealed (so that assignments could not be predicted)?
Q4. Were study participants and providers blinded to treatment group assignment?
Q5. Were the people assessing the outcomes blinded to the participants' group assignments?
Q6. Were the groups similar at baseline on important characteristics that could affect outcomes (e.g., demographics, risk factors, co-morbid conditions)?
Q7. Was the overall drop-out rate from the study at endpoint 20% or lower of the number allocated to treatment?
Q8. Was the differential drop-out rate (between treatment groups) at endpoint 15 percentage points or lower?
Q9. Was there high adherence to the intervention protocols for each treatment group?
Q10. Were other interventions avoided or similar in the groups (e.g., similar background treatments)?
Q11. Were outcomes assessed using valid and reliable measures, implemented consistently across all study participants?
Q12. Did the authors report that the sample size was sufficiently large to be able to detect a difference in the main outcome between groups with at least 80% power?
Q13. Were outcomes reported or subgroups analyzed prespecified (i.e., identified before analyses were conducted)?
Q14. Were all randomized participants analyzed in the group to which they were originally assigned, i.e., did they use an intention-to-treat analysis?

*CD, cannot determine; NA, not applicable; NR, not reported

© 2025 Byiringiro S et al. *JAMA Network Open*.

eTable 3. Weighted Levels of FQHC Engagement by the Clinical Trials' Quality Assessment and Start Year

	Level 1, n= 19	Level 2, n= 38	Level 3 or 4, n= 10	Overall, n=67
**Quality Assessment of the Studies**				
Good or Fair	15 (21.7%)	6 (9.3%)	8 (12.4%)	29 (43.4%)
Poor	6 (9.3%)	6 (9.3%)	7 (10.1%)	19 (28.7%)
Protocol (no rating)	11 (16%)	4 (6.2%)	4 (6.2%)	19 (27.9%)
**Intervention start year, mean, (95% CI)**	2016 (2014, 2018)	2014 (2012, 2016)	2014 (2012, 2016)	2015 (2013, 2016)

eTable 4. Results of Weighted Ordinal Regression Models of Levels of FQHC Engagement and the Quality Assessment and Intervention Start Year

FQHC Characteristic	Unadjusted (OR 95%CI)	Adjusted (OR 95%CI)[1]
**Quality of the study**		
Good or Fair	**Ref.**	**Ref.**
Poor	1.71 (0.35, 8.27)	1.53 (0.27, 8.82)
Protocol (no rating)	0.77 (0.14, 4.33)	1.65 (0.15, 17.83)
**Intervention start year**	0.879 (0.72, 1.07)	0.87 (0.68, 1.12)

Abbreviation: FTE - Full-time Equivalents; FQHC – Federally Qualified Community Health Centers; OR – Odds Ratio; CI – Confidence Interval

* p<0.05 | ** p≤0.01

[†]Odds of higher level of engagement associated with every 1-unit increase in FTE per 10,000 adult patients served annually

[1]Adjusted for disease type, location, and both included variables.

© 2025 Byiringiro S et al. *JAMA Network Open*.

eFigure 1. Data Sources and Process for Data Acquisition

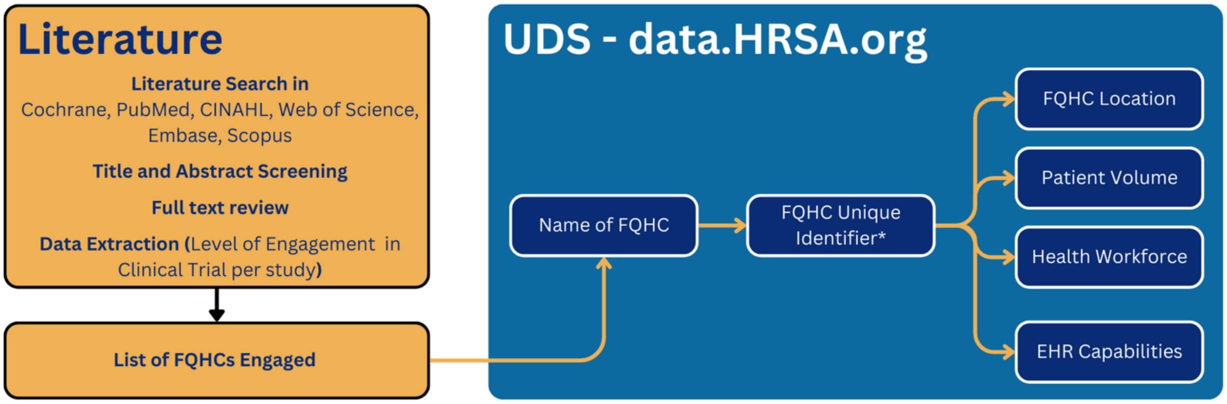

Abbreviation: FQHC - Federally Qualified Health Center, UDS - Uniform Data System, EHR: Electronic Health Record. * We looked up and downloaded the FQHC data of the year in which the clinical trial was initiated (FQHC was engaged).

Note: Figure created by authors.

eFigure 2. Levels of FQHC Engagement in Clinical Trials

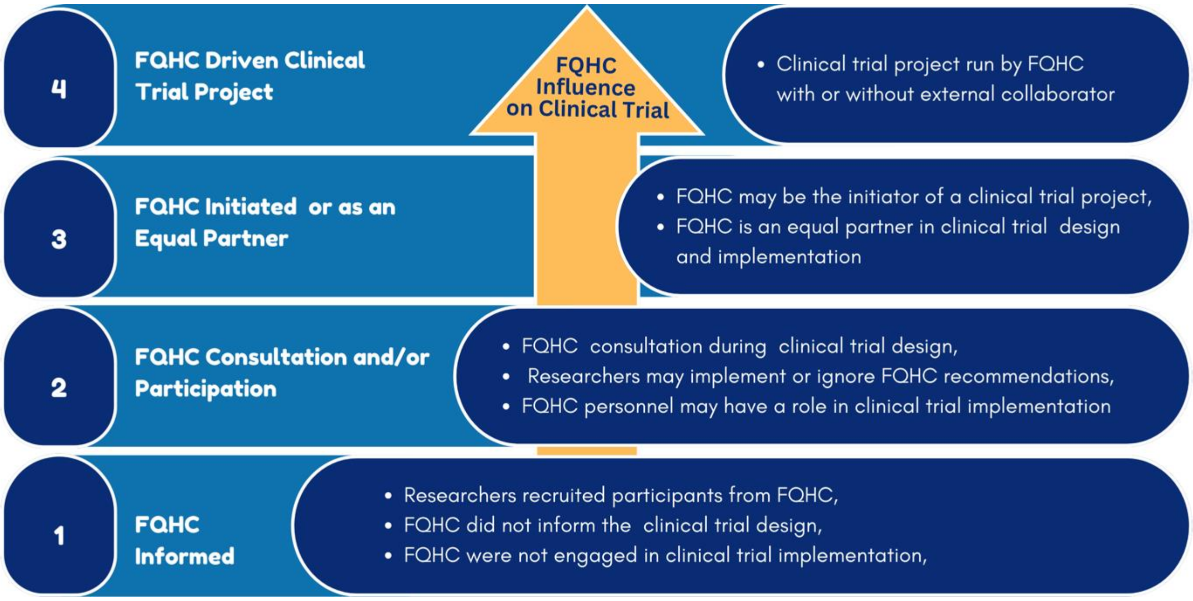

Note: Figure created by authors. **Abbreviation:** FQHC - Federally Qualified Health Center

© 2025 Byiringiro S et al. *JAMA Network Open*.

eFigure 3. Location of and FQHCs Engaged in Hypertension and Type 2 Diabetes Clinical Trials

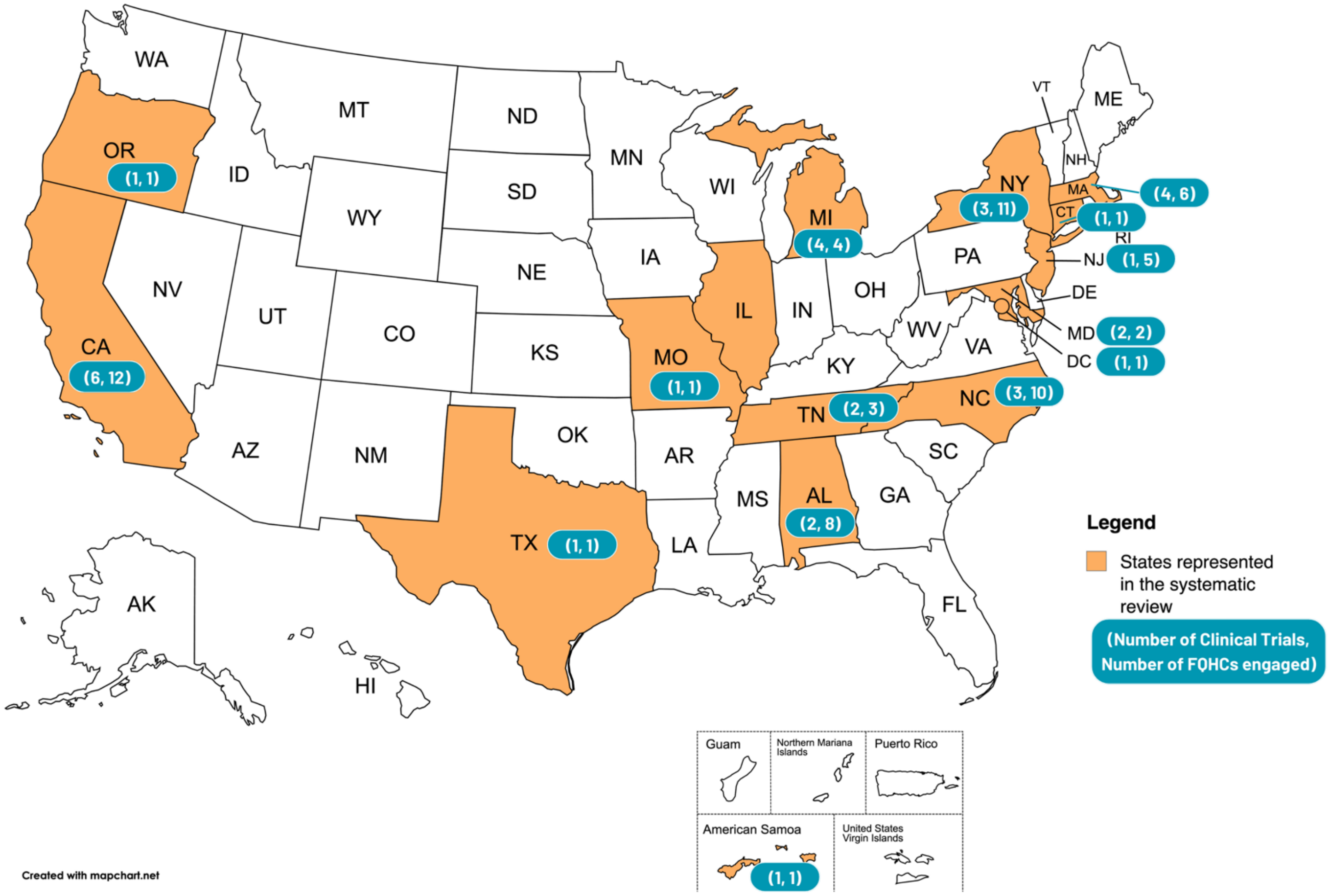

Note: Map created with CC BY-SA 4.0 permission from MapChats: https://www.mapchart.net/terms.html#licensing-maps.
Abbreviation: FQHC - Federally Qualified Health Center

© 2025 Byiringiro S et al. *JAMA Network Open*.

eFigure 4. Levels of FQHC Engagement in Clinical Trials Over Intervention Start Years

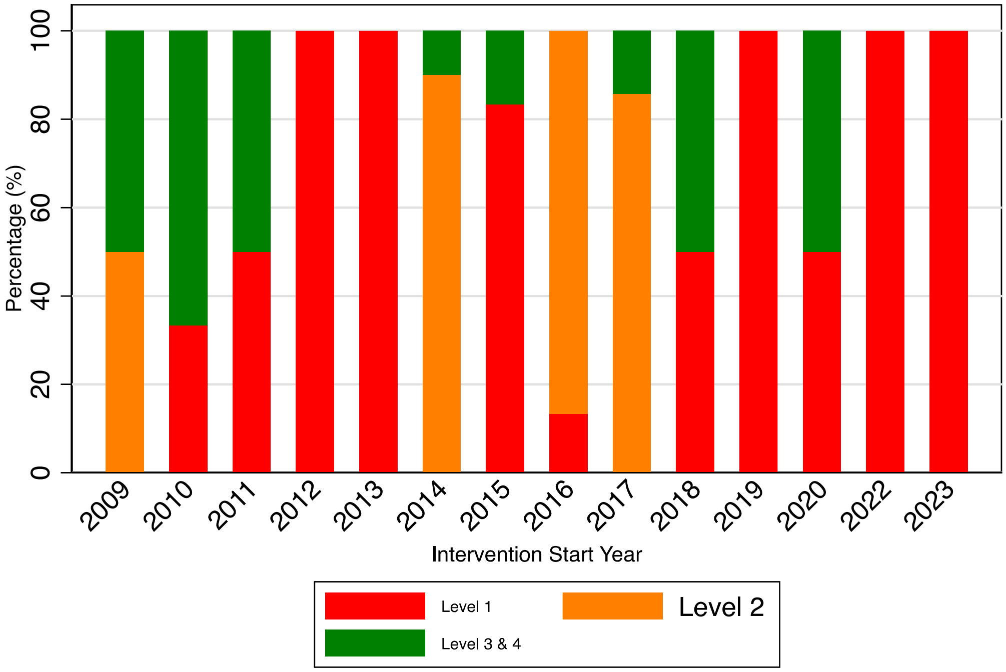

Notes: Levels of FQHC engagement: Level - 1) Did not engage FQHC in intervention design, Level - 2) Elicited FQHC feedback during study design, Level - 3) Project conceived by or co-developed and implemented in partnership with FQHC, and Level - 4) Project developed and conducted by a FQHC. **Abbreviation:** FQHC - Federally Qualified Health Center

© 2025 Byiringiro S et al. *JAMA Network Open*.

eReferences.

1. Study Quality Assessment Tools | NHLBI, NIH. Accessed August 25, 2024. https://www.nhlbi.nih.gov/health-topics/study-quality-assessment-tools

2. Key KD, Furr-Holden D, Yvonne Lewis E, et al. The Continuum of Community Engagement in Research: A Roadmap for Understanding and Assessing Progress. *Prog Community Health Partnersh*. 2019;13(4):427-434. doi:10.1353/CPR.2019.0064

3. Li F, Morgan KL, Zaslavsky AM. Balancing Covariates via Propensity Score Weighting. *J Am Stat Assoc*. 2018;113(521):390-400. doi:10.1080/01621459.2016.1260466

4. Williams R. Understanding and interpreting generalized ordered logit models. *J Math Sociol*. 2016;40(1):7-20. doi:10.1080/0022250X.2015.1112384

5. Williams R, Williams RA 5@nd E. gologit2 documentation. Published online 2007. Accessed January 15, 2025. http://www.nd.edu/~rwilliam/gologit2/index.htmlExamples:.usehttp://www.indiana.edu/~jslsoc/stata/spex_data/ordwarm2.dta,clear.sw,pe

6. Williams R. Generalized Ordered Logit/Partial Proportional Odds Models for Ordinal Dependent Variables. *https://doi.org/101177/1536867X0600600104*. 2006;6(1):58-82. doi:10.1177/1536867X0600600104

7. Bluml BM, Kolb LE, Lipman R. Evaluating the impact of year-long, augmented diabetes self-management support. *Popul Heath Manage*. 2019;22(6):522-528. doi:10.1089/pop.2018.0175

8. Bryce R, WolfsonBryce JA, CohenBryce A, et al. A pilot randomized controlled trial of a fruit and vegetable prescription program at a federally qualified health center in low income uncontrolled diabetics. *Preventive Med Reports*. 2021;23. doi:10.1016/j.pmedr.2021.101410

9. Clark TL, Gallo L, Euyoque JA, Philis-Tsimikas A, Fortmann A. Does Diabetes Distress Influence Clinical Response to an mHealth Diabetes Self-Management Education and Support Intervention? *Diabetes Educ*. 2020;46(3):289-296. doi:10.1177/0145721720913276

10. Commodore-Mensah Y, Liu X, Ogungbe O, et al. Design and Rationale of the Home Blood Pressure Telemonitoring Linked with Community Health Workers to Improve Blood Pressure (LINKED-BP) Program. *Am J Hypertens*. 2023;36(5):273-282-. doi:10.1093/ajh/hpad001

11. Delahanty LM, Chang YH, Levy DE, et al. Design and participant characteristics of a primary care adaptation of the Look AHEAD Lifestyle Intervention for weight loss in type 2 diabetes: The updates REAL HEALTH-diabetes study. *Contemp Clin Trials*. 2018;71:9-17. doi:10.1016/j.cct.2018.05.018

12. De Pue JD, Dunsiger S, Seiden AD, et al. Nurse-community health worker team improves diabetes care in American Samoa: Results of a randomized controlled trial. *Diabetes Care*. 2013;36(7):1947-1953. doi:10.2337/dc12-1969

13. Deverts DJ, Heisler M, Kieffer EC, et al. Comparing the effectiveness of Family Support for Health Action (FAM-ACT) with traditional community health worker-led interventions to improve adult diabetes management and outcomes: study protocol for a randomized controlled trial. *Trials*. 2022;23(1):841. doi:10.1186/s13063-022-06764-1

14. Dodson JA, Schoenthaler A, Fonceva A, et al. Study design of BETTER-BP: Behavioral economics trial to enhance regulation of blood pressure. *International Journal of Cardiology: Cardiovascular Risk and Prevention*. 2022;15. doi:10.1016/j.ijcrp.2022.200156

© 2025 Byiringiro S et al. *JAMA Network Open*.

15. Fiscella K, He H, Sanders M, et al. Blood Pressure Visit Intensification in Treatment (BP-Visit) Findings: a Pragmatic Stepped Wedge Cluster Randomized Trial. *J Gen Intern Med*. 2022;37(1):32-39. doi:10.1007/s11606-021-07016-9

16. Garrison TA, Schwartz JK, Moore ES. Effect of Occupational Therapy in Promoting Medication Adherence in Primary Care: A Randomized Controlled Trial. *Am J Occup Ther*. 2023;77(3). doi:10.5014/ajot.2023.050109

17. Hargraves JL, Bonollo D, Person SD, Ferguson WJ. A randomized controlled trial of community health workers using patient stories to support hypertension management: Study protocol. *Contemp Clin Trials*. 2018;69:76-82. doi:10.1016/j.cct.2018.04.004

18. Heisler M, Choi H, Palmisano G, et al. Comparison of Community Health Worker-Led Diabetes Medication Decision-Making Support for Low-Income Latino and African American Adults With Diabetes Using E-Health Tools Versus Print Materials A Randomized, Controlled Trial. *Ann Intern Med*. 2014;161(10):S13-S22. doi:10.7326/m13-3012

19. Heitkemper EM, Mamykina L, Tobin JN, Cassells A, Smaldone A. Baseline Characteristics and Technology Training of Underserved Adults With Type 2 Diabetes in the Mobile Diabetes Detective (MoDD) Randomized Controlled Trial. *Diabetes Educ*. 2017;43(6):576-588. doi:10.1177/0145721717737367

20. Hessler D, Fisher L, Dickinson M, Dickinson P, Parra J, Potter MB. The impact of enhancing self-management support for diabetes in Community Health Centers through patient engagement and relationship building: a primary care pragmatic cluster-randomized trial. *Transl Behav Med*. 2022;12(9):909-918. doi:10.1093/tbm/ibac046

21. Ibe CA, Haywood DR, Creighton C, et al. Study protocol of a randomized controlled trial evaluating the Prime Time Sister Circles (PTSC) program's impact on hypertension among midlife African American women. *BMC Public Health*. 2021;21(1):610. doi:10.1186/s12889-021-10459-8

22. Kahlon. Empathy in Action: sunshine Calls for Life With Diabetes. *https://clinicaltrials.gov/show/NCT05173675*. Published online 2021. https://www.cochranelibrary.com/central/doi/10.1002/central/CN-02354235/full

23. Khanna R, Stoddard PJ, Gonzales EN, et al. An automated telephone nutrition support system for Spanish-speaking patients with diabetes. *J Diabetes Sci Technol*. 2014;8(6):1115-1120. doi:10.1177/1932296814550186

24. Koonce TY, Giuse NB, Kusnoor S V, Hurley S, Fei Y. A personalized approach to deliver health care information to diabetic patients in community care clinics. *Journal of the Medical Library Association*. 2015;103(3):123-130. doi:10.3163/1536-5050.103.3.004

25. Lindberg NM, Vega-López S, LeBlanc ES, et al. Lessons Learned From a Program to Reduce Diabetes Risk Among Low-Income Hispanic Women in a Community Health Clinic. *Front Endocrinol*. 2021;11. doi:10.3389/fendo.2020.489882

26. Mitchell SE, Bragg A, de la Cruz BA, et al. Effectiveness of an Immersive Telemedicine Platform for Delivering Diabetes Medical Group Visits for African American, Black and Hispanic, or Latina Women With Uncontrolled Diabetes: The Women in Control 2.0 Noninferiority Randomized Clinical Trial. *J Med Internet Res*. 2023;25:16. doi:10.2196/43669

27. Nelson LA, Wallston KA, Kripalani S, et al. Mobile phone support for diabetes self-care among diverse adults: Protocol for a three-arm randomized controlled trial. *JMIR Res Prot*. 2018;7(4). doi:10.2196/resprot.9443

28. Persell SD, Karmali KN, Lazar D, et al. Effect of Electronic Health Record-Based Medication Support and Nurse-Led Medication Therapy Management on Hypertension and Medication Self-management: a Randomized Clinical Trial. *JAMA Intern Med*. 2018;178(8):1069-1077-. doi:10.1001/jamainternmed.2018.2372

© 2025 Byiringiro S et al. *JAMA Network Open*.

29. Philis-Tsimikas A, Fortmann AL, Godino JG, et al. Dulce Digital-Me: protocol for a randomized controlled trial of an adaptive mHealth intervention for underserved Hispanics with diabetes. *Trials*. 2022;23(1). doi:10.1186/s13063-021-05899-x

30. Presley. Mindfulness-Based Diabetes Education for Adults With Elevated Diabetes Distress. *https://clinicaltrials.gov/show/NCT05195138*. Published online 2022. https://www.cochranelibrary.com/central/doi/10.1002/central/CN-02366877/full

31. Redmond ML, Nollen N, Okut H, et al. eDECIDE a web-based problem-solving interventions for diabetes self-management: Protocol for a pilot clinical trial. *Contemp Clin Trials Comm*. 2023;32. doi:10.1016/j.conctc.2023.101087

32. Shapiro MF, Shu SB, Goldstein NJ, et al. Impact of a Patient-Centered Behavioral Economics Intervention on Hypertension Control in a Highly Disadvantaged Population: a Randomized Trial. *JGIM: Journal of General Internal Medicine*. 2020;35(1):70-78. doi:10.1007/s11606-019-05269-z

33. Shikany JM, Safford MM, Cherrington AL, et al. Recruitment and retention of primary care practices in the Southeastern Collaboration to Improve Blood Pressure Control. *Contemp Clin Trials Commun*. 2023;32. doi:10.1016/j.conctc.2023.101059

34. Smith. Short Messaging Service for Optimizing Hemoglobin A1C Management in Low-Income Diabetics. *https://clinicaltrials.gov/show/NCT02049359*. Published online 2014. https://www.cochranelibrary.com/central/doi/10.1002/central/CN-01543329/full

35. Spencer MS, Kieffer EC, Sinco B, et al. Outcomes at 18 Months From a Community Health Worker and Peer Leader Diabetes Self-Management Program for Latino Adults. *Diabetes Care*. 2018;41(7):1414-1422-. doi:10.2337/dc17-0978

36. Steinberg D, Kay M, Burroughs J, Svetkey LP, Bennett GG. The Effect of a Digital Behavioral Weight Loss Intervention on Adherence to the Dietary Approaches to Stop Hypertension (DASH) Dietary Pattern in Medically Vulnerable Primary Care Patients: Results from a Randomized Controlled Trial. *J Acad Nutri Diet*. 2019;119(4):574-584. doi:10.1016/j.jand.2018.12.011

37. Thom DH, Ghorob A, Hessler D, De Vore D, Chen E, Bodenheimer TA. Impact of peer health coaching on glycemic control in low-income patients with diabetes: A randomized controlled trial. *Ann Fam Med*. 2013;11(2):137-144. doi:10.1370/afm.1443

38. Van Name MA, Camp AW, Magenheimer EA, et al. Effective Translation of an Intensive Lifestyle Intervention for Hispanic Women With Prediabetes in a Community Health Center Setting. *Diabetes Care*. 2016;39(4):525-531. doi:10.2337/dc15-1899

39. Welch G, Zagarins SE, Santiago-Kelly P, et al. An internet-based diabetes management platform improves team care and outcomes in an urban Latino population. *Diabetes Care*. 2015;38(4):561-567. doi:10.2337/dc14-1412

© 2025 Byiringiro S et al. *JAMA Network Open*.
