# Supplementary material for: Engagement in Hypertension and Diabetes Clinical Trials at Federally Qualified Health Centers: A Systematic Review
Source: JAMA Netw Open. 2025 Apr 15;8(4):e255258. doi: 10.1001/jamanetworkopen.2025.5258 (PMC12000987; doi:10.1001/jamanetworkopen.2025.5258)
Supplement: Supplement 2. — Data Sharing Statement [file jamanetwopen-e255258-s002.pdf]

Data Sharing Statement

Byiringiro. Engagement in Hypertension and Diabetes Clinical Trials at Federally Qualified Health Centers. *JAMA Netw Open*. Published April 15, 2025.
doi:10.1001/jamanetworkopen.2025.5258

Data
Data available: No

Additional Information
Explanation for why data not available: All data used are publicly available. For reproducibility, a thorough description of the methods is provided in the article and supplement 1.
